# First Detection of DS-1-like G1P[8] Double-gene Reassortant Rotavirus Strains on The American Continent, Brazil, 2013

**DOI:** 10.1038/s41598-019-38703-7

**Published:** 2019-02-18

**Authors:** Adriana Luchs, Antonio Charlys da Costa, Audrey Cilli, Shirley Cavalcante Vasconcelos Komninakis, Rita de Cássia Compagnoli Carmona, Simone Guadagnucci Morillo, Ester Cerdeira Sabino, Maria do Carmo Sampaio Tavares Timenetsky

**Affiliations:** 1 Enteric Disease Laboratory, Virology Center, Adolfo Lutz Institute, São Paulo, Brazil; 2 LIM/46 - Laboratory of Medical Parasitology, Department of Infectious and Parasitic Diseases, College of Medicine, University of São Paulo, São Paulo, Brazil; 3 Institute of Tropical Medicine, University of São Paulo, São Paulo, Brazil; 4 Postgraduate Program in Health Science, Faculty of Medicine of ABC, Santo André, Brazil; 5 Retrovirology Laboratory, Federal University of São Paulo, São Paulo, São Paulo, Brazil

## Abstract

Emergence of DS-1-like-G1P[8] rotavirus in Asia have been recently reported. We report for the first time the detection and the whole genome phylogenetic analysis of DS-1-like-G1P[8] strains in America. From 2013 to 2017, a total of 4226 fecal samples were screened for rotavirus by ELISA, PAGE, RT-PCR and sequencing. G1P[8] represented 3.7% (30/800) of all rotavirus-positive samples. DS-1-like-G1P[8] comprised 1.6% (13/800) detected exclusively in 2013, and Wa-like-G1P[8] comprised 2.1% (17/800) detected from 2013 to 2015. Whole genome sequencing confirmed the DS-1-like backbone I2-R2-C2-M2-A2-N2-T2-E2-H2. All genome segments of the Brazilian DS-1-like-G1P[8] strains clustered with those of Asian strains, and apart from African DS-1-like-G1P[8] strains. In addition, Brazilian DS-1-like-G1P[8] reassortants distantly clustered with DS-1-like backbone strains simultaneously circulating in the country, suggesting that the Brazilian DS-1-like-G1P[8] strains are likely imported from Asia. Two distinct NSP4 E2 genotype lineages were also identified, indicating the existence of a co-circulating pool of different DS-1-like G1P[8] strains. Surveillance systems must be developed to examine if RVA vaccines are still effective for the prevention against unusual DS-1-like-G1P[8] strains.

## Introduction

Rotavirus (RVA) is the major etiologic agent of acute gastroenteritis in young children worldwide. Rotarix^TM^ vaccine (GlaxoSmithKline Biologicals, Belgium) was included in the Brazilian Immunization Program in 2006 and has had an excellent acceptance in subsequent years and resulted in a significant decline in the RVA disease burden^1–3^. The majority of human RVA genomes are assigned to three genotype constellations: Wa-like or genogroup 1 (G1/3/4/9/12-P[8]-I1-R1-C1-M1-A1-N1-T1-E1-H1), DS-1-like or genogroup 2 (G2-P[4]-I2-R2-C2-M2-A2-N2-T2-E2-H2) and AU-1-like or genogroup 3 (G3-P[9]-I3-R3-C3-M3-A3-N3-T3-E3-H3)^4^. Intergenogroup reassortant strains can be generated; however, such RVA strains possessing both Wa-like and DS-1-like genes exhibit reduced evolutionary fitness and, therefore, would be selected against in nature^5^. Nevertheless, the emergence of novel previously undescribed human intergenogroup reassortant strains, DS-1-like G1P[8] and G3P[8] strains, were recently reported in different parts of the world^6–16^.

During the 2013 National RVA surveillance, unusual strains of the G1P[8] genotype displaying a short electropherotype pattern were identified. A general correlation between G/P type and RNA profile has been observed: G1/3/4/9/12 P[8] (Wa-like constellation) shows a long RNA pattern, while G2P[4] (DS-1-like constellation) exhibits a short RNA pattern^4,10^. The short electropherotype profile associated with the G1P[8] genotype could be a guide mark to the identification of the emerging DS-1-like G1P[8] RVA strain. These G1P[8] DS-1-like strains emerged in 2012 and appeared to be restricted to Asia^10–13^. Nevertheless, G1P[8] DS-1-like strains have been recently reported in Malawi, Africa^14^.

The objectives of the present study were to report the first detection of G1P[8] DS-1-like strains in the Americas and to characterize their genomes. In addition, phylogenetic analysis was conducted in order to understand their genetic diversity and evolution.

## Materials and Methods

### Study specimens and RVA detection

This is a retrospective and descriptive study conducted with clinical samples. Brazil is a continental-size country, and the Brazilian Rotavirus Surveillance Program is funded among three collaborating institutes: (i) Evandro Chagas Institute, national and regional reference center for RVA surveillance in the Northern region and part of the Northeastern region; (ii) Oswaldo Cruz Institute, regional reference center for RVA surveillance in part of the Northeastern, Southeastern and Southern regions; and (iii) Adolfo Lutz Institute, regional reference center for RVA surveillance in Midwest and part of the Southeastern and Southern regions.

During the period January 2013 to December 2017, a total of 4226 fecal samples were collected from children and adults with acute gastroenteritis for analysis at collaborating centers across Southern, Southeastern and Midwest Brazil, located in the states of São Paulo (n = 3183), Mato Grosso (n = 33), Mato Grosso do Sul (n = 113), Paraná (n = 408), and Goiás (including the Federal District) (n = 489). They were then sent to the Enteric Diseases Laboratory of the Adolfo Lutz Institute (Central Public Health Laboratory of São Paulo State). Thus, the sample collection is likely to be representative of Brazilian population. All specimens were screened for RVA by a commercial enzyme-linked immunosorbent assay (ELISA) (Ridascreen®Rotavirus, R-biopharm, Darmstadt, Germany), according to the manufacturer´s protocol. RVA positive specimens detected by ELISA in the collaborating laboratories located in the states of Mato Grosso, Mato Grosso do Sul, Paraná and Goiás were stored frozen and forwarded to the Enteric Diseases Laboratory of the Adolfo Lutz Institute for RVA genotyping, together with relevant age and sex data. The participating laboratories also sent limited amount of negative RVA samples detected during the surveillance as part of the supervised quality program. The Enteric Diseases Laboratory of the Adolfo Lutz Institute conducted the RVA surveillance in the State of São Paulo.

### Electropherotyping, viral RNA extraction, G-/P- genotyping, and VP7 sequencing

The RVA migration profiles were analyzed by PAGE, followed by silver staining of gels^17^. Viral RNA was extracted from 10% fecal extracts using PureLink^TM^ RNA Mini kit (Invitrogen®, Life Technologies, Carlsbad, CA, USA), according to the manufacturer’s instructions, and subjected to G and P typing by multiplex reverse transcription-polymerase chain reaction (RT-PCR) with type-specific primers, following previously described protocols^18–21^. First round amplicons of G1 VP7 RVA positive samples were selected for sequencing. PCR amplicons were sequenced using the BigDye Terminator v3.1 Cycle Sequencing Kit (Applied Biosystems, Foster City, CA, USA) with primers Beg9/End9 (1062 bp). Dye-labelled products were sequenced in an ABI 3130 DNA Analyzer (Applied Biosystems, Foster City, CA, USA). Sequences were edited with Sequencher 4.7 software. The genome assignment was accomplished using the web-based automated RVA genotyping tool RotaC2.0 (http://rotac.regatools.be) in order to confirm the detected G1 genotype^22^. The obtained G1 sequences were also individually compared with RVA sequences available in GenBank. When G1P[8] DS-1-like reassortment was suspected based on RNA profile, the most similar sequences available in GenBank were also added to the corresponding tree together with classical, well characterized G1P[8] Wa-like strains. Phylogenetic analysis was performed using the software MEGA 7.0^23^. Phylogenetic trees were constructed using the Neighbour-Joining method and Kimura’s two-parameter model with 1000 bootstrap replicates. Therefore, suspected G1P[8] DS-1-like strains could be identified on the basis of preliminary phylogenetic analysis together with RNA pattern analysis.

### Nucleotide sequencing of the complete DS-1-like G1P[8] RVA genomes

Based on Brazilian regional location and sample availability, four atypical DS-1-like G1P[8] RVA strains with short RNA migration patterns were selected for investigation of the whole genome. These include three DS-1-like G1P[8] strains (RVA/Human-wt/BRA/IAL-R3122/2013/G1P[8], RVA/Human-wt/BRA/IAL-R3123/2013/G1P[8] and RVA/Human-wt/BRA/IAL-R3165/2013/G1P[8]) detected in the state of São Paulo in 2013 and one DS-1-like G1P[8] strain (RVA/Human-wt/BRA/IAL-R3172/2013/G1P[8]) detected in the state of Goiás in the same year.

The protocol used to perform deep-sequencing was a combination of several protocols normally applied to viral metagenomics and/or virus discovery and has been partially described by Charlys da Costa *et al*.^24^. In summary, 50 mg of the human fecal samples were diluted in 500 μl of Hanks’ buffered salt solution (HBSS) added to a 2 ml impact-resistant tube containing lysing matrix C (MP Biomedicals, USA) and homogenized in a FastPrep-24 5 G Homogenizer (MP biomedicals, USA). The homogenized sample was centrifuged at 12,000 × *g* for 10 min, and approximately 300 μl of the supernatant was then percolated through a 0.45 μm filter (Merck Millipore, Billerica, MA, USA) in order to remove eukaryotic and bacterial cell-sized particles. Roughly the equivalent of one fourth of the volume of the tube of cold PEG-it Virus Precipitation Solution (System Biosciences, CA, USA), approximately 100 uL, was added to the obtained filtrate, the contents of the tube were gently mixed, and then they were incubated at 4 °C for 24 hours. After the incubation period, the mixture was centrifuged at 10000 × *g* for 30 minutes at 4 °C. Following centrifugation, the supernatant (~350 μl) was discarded. The pellet, rich in viral particles, was treated with a combination of nuclease enzymes (TURBO DNase and RNase Cocktail Enzyme Mix - Thermo Fischer Scientific, CA, USA; Baseline-ZERO DNase - Epicentre, WI, USA; Benzonase - Darmstadt, Germany; and RQ1 RNase-Free DNase and RNase A Solution - Promega, WI, USA) in order to digest unprotected nucleic acids. The resulting mixture was subsequently incubated at 37 °C for 2 h.

After incubation, viral nucleic acids were extracted using ZR & ZR-96 Viral DNA/RNA Kit (Zymo Research, CA, USA) according to the manufacturer’s protocol. The cDNA synthesis was performed with AMV Reverse transcription (Promega, WI, USA). A second strand of cDNA synthesis was performed using DNA Polymerase I Large (Klenow) Fragment (Promega, WI, USA). Subsequently, a Nextera XT Sample Preparation Kit (Illumina, CA, USA) was used to construct a DNA library, identified using dual barcodes. For size range, Pippin Prep (Sage Science, Inc.) was used to select a 300 bp insert (range 200–400 bp). The library was deep-sequenced using the Hi-Seq. 2500 Sequencer (Illumina, CA, USA) with 126 bp ends. Bioinformatic analysis was performed according to the protocol previously described by Deng *et al*.^25^. Contigs that shared a percent nucleotide identity of 95% or less were assembled from the obtained sequence reads by *de novo* assembly. The contigs included the group A rotavirus sequences and others, such as enteric viruses, human, fungal, and bacterial sequences. The different titers of RVA genomic RNA extracted from the stool specimen will reflect the number of copies in the library, and therefore, the number of reads obtained for the genes of each strain. After identification of RVA in each sample, a reference template sequence was used for mapping the full-length genome with Geneious R9^26^. The complete or nearly complete nucleotide sequence of each gene segment of the 11 DS-1-like G1P[8] strain was obtained by using BLAST against local data in Sequencher 4.7 software with the assembled contigs as query sequences and 11 genome segments of reference RVA as the target sequence.

### Phylogenetic analysis

The genotype of each genome segment was classified by using the RotaC2.0 automated genotyping tool for RVA (http://rotac.regatools.be)^22^. The nucleotide sequences available from GenBank were compiled using the BLAST program (http://blast.ncbi.nlm.nih.gov) with the sequences of the herein described DS-1-like G1P[8] strains as the query sequences for VP1-4, VP6-7 and NSP1-4, 5/6 genome segments. The nearly full-length genomes sequences describe in this study were aligned with the sequences obtained from the GenBank database using the CLUSTAL W algorithm in BioEdit Sequence Alignment Editor (version 7.0.5.2) Program^27^. Nucleotide and amino acid distance matrixes were calculated using a p-distance algorithm in MEGA 7.0^23^. A maximum likelihood tree was constructed for each genome segment. The best substitution models were selected based on the corrected Akaike Information Criterion (AICc) value as implemented in MEGA 7.0. The models used in this study were Tamura 3-parameter (T92) + G (NSP2, NSP3, NSP4, NSP5, VP3, VP4, and VP7), T92 + I (NSP1 and VP6), Hasegawa-Kishino-Yano (HKY) + G (VP1), and Tamura-Nei (TN93) + G (VP2). The statistical significance at the branch point was calculated with 1000 pseudo-replicate datasets.

Nucleotide sequences determined in this study have been deposited in GenBank under the accession numbers MG599515-MG599538 for the VP1-4, 6 and 7 genes, and MG573355-MG573374 for the NSP1-4 and 5/6 genes.

### Ethical approval

Previous Ethics Committee approval was granted by the Adolfo Lutz Institute, São Paulo, Brazil (CEP 965.723; CTC 45G-2014). This was an anonymous unlinked study and informed consent was not required according to resolution 466/12 concerning research involving humans (Conselho Nacional de Saúde/Ministério da Saúde, Brasília, 2012).

## Results

The G1P[8] genotype represented 3.7% (30/800) of all RVA-positive samples. All G1 VP7 amplicons were successfully sequenced and generate a reliable classification as Wa-like or DS-1-like. These were further differentiated as DS-1-like G1P[8] (1.6%; 13/800) and Wa-like G1P[8] (2.1%; 17/800) after confirmatory G1 VP7 sequencing and RNA pattern analysis. All Wa-like G1P[8] strains showed the typical long RNA pattern, while all DS-1-like G1P[8] RVA specimens exhibited a short RNA migration profile (Supplement 1). Distribution of RVA genotypes detected during the study period were reported in previous investigations^9,28^. The atypical DS-1-like G1P[8] strain was first identified in August 2013 in a 2 year-old non-vaccinated female from the city of São Paulo, State of São Paulo. During 2013, the Wa-like G1P[8] genotype (51.8%; 14/27) and DS-1-like G1P[8] strain (48.2%; 13/27) were concomitantly detected. However, the DS-1-like G1P[8] strains were mostly identified in the State of São Paulo (84.6%; 11/13) and only two DS-1-like strains were detected in the State of Goiás (15.4%; 2/13). During 2014 and 2015, only Wa-like G1P[8] strains were detected (100%; 3/3), and no G1P[8] strain was detected in 2016 and 2017 (Supplement 2).

Complete or nearly complete nucleotide sequences for 11 genome segments of the four DS-1-like G1P[8] strains were determined using the NGS (next generation sequencing) Illumina Hi-Seq platform. The length and related read data of the obtained sequences are shown in Supplements 3 and 4. All four DS-1-like G1P[8] strains (RVA/Human-wt/BRA/IAL-R3122/2013/G1P[8], RVA/Human-wt/BRA/IAL-R3123/2013/G1P[8], RVA/Human-wt/BRA/IAL-R3165/2013/G1P[8] and RVA/Human-wt/BRA/IAL-R3172/2013/G1P[8]) were double-reassortant strains carrying G1-VP7 and P[8]-VP4 genes on a DS-1-like genetic background (I2-R2-C2-M2-A2-N2-T2-E6-H2) (Supplement 5).

To investigate the genetic relatedness and possible origin of the Brazilian DS-1-like G1P[8] strains, the 11 gene segments were analyzed phylogenetically. The phylogenetic relationship was inferred using the Maximum-Likelihood method with complete (when available) RVA gene segments. For the designation of lineages, strains from GenBank were selected using the VP7 and VP4 lineages previously published by Arista *et al*.^29^, and DS-1-like backbone formerly suggested by Doan *et al*.^30^ and Aida *et al*.^31^. Sequences of Wa-like G1 and P[8], and DS-1- like backbone strains from Brazil and South America were also included. It is worth mentioning, that an important limitation of the Brazilian RVA genomics study is that complete genome sequences are not generally available. Although several Brazilian studies have already been conducted with full genomic constellation analysis^32,33^, they usually focus on complete genotype classification with partial ORF sequences. Therefore, the full genome sequences of the larger RVA segments (e.i. VP1, VP2, VP3 and VP4) are mostly unavailable.

The nucleic acid identities of the strains of this study in relation to the ones used for the phylogenetic analysis are shown in Supplement 6. The VP1-4, VP6-7 and NSP1-5/6 genes from the four Brazilian DS-1-like G1P[8] strains (RVA/Human-wt/BRA/IAL-R3122/2013/G1P[8], RVA/Human-wt/BRA/IAL-R3123/2013/G1P[8], RVA/Human-wt/BRA/IAL-R3165/2013/G1P[8] and RVA/Human-wt/BRA/IAL-R3172/2013/G1P[8]) exhibited high level sequence conservation with >99% sequence identity to each other. Percentages of nt identity between Brazilian DS-1-like G1P[8] to other DS-1like G1P[8] strains varied across DS-1-like backbone RVA gene segments: 96.4–99.8% for NSP1, 91.7–98.4% for NSP2, 92.7–99.8% for NSP3, 84.8–98.5% for NSP4, 89.0–99.3% for NSP5, 93.7–99.4% for VP1, 97.2–99.7% for VP2, 95.8–98.9% for VP3, and 93.8–99.0% for VP6. Similar nt sequence identities (97.4–99.9%) were also observed between Brazilian DS-1-like G1P[8] strains detected here and DS-1-like backbone genes from equine like G3P[8] DS-1-like strains detected worldwide.

**Figure 1:**
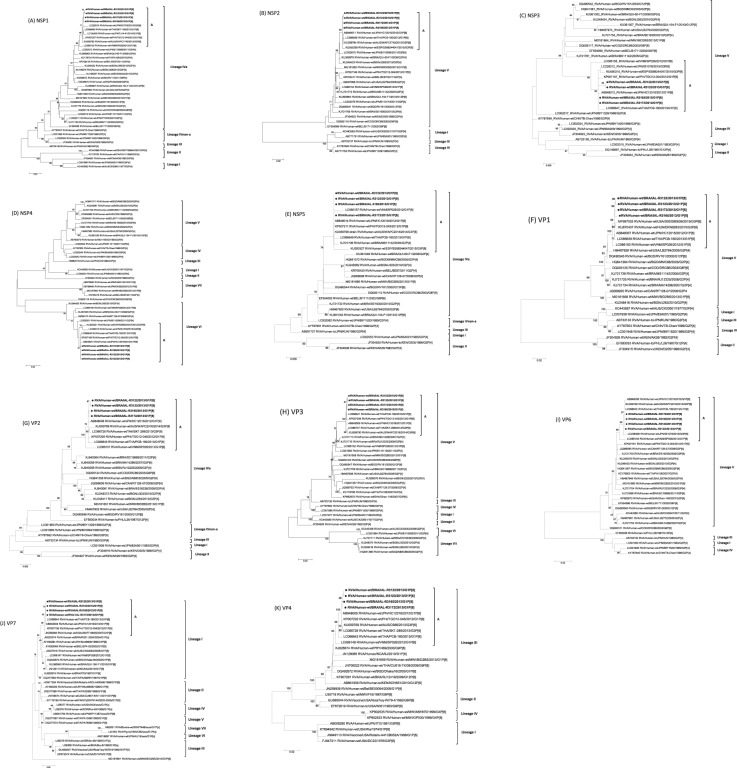
Nucleotide based phylogenetic relatedness of RVA/Human-wt/BRA/IAL-R3122/2013/G1P[8], RVA/Human-wt/BRA/IAL-R3123/2013/G1P[8], RVA/Human-wt/BRA/IAL-R3165/2013/G1P[8], and RVA/Human-wt/BRA/IAL-R3172/2013/G1P[8] RVA genes (indicated in bold and by ●) (**A**) NSP1, (**B**) NSP2, (**C**) NSP3, (**D**) NSP4, (**E**) NSP5/6, (**F**) VP1, (**G**) VP2, (**H**) VP3, (**I**) VP6, (**J**) VP7 and (**K**) VP4 to other selected RVA strains. Maximum likelihood trees of complete nucleotide sequences were generated with MEGA 7.0 software. Reference strains were obtained from GenBank database. Genotypes, lineages, accession number, isolates, countries and year of each strain are indicated. The scale indicates the number of divergent nucleotide residues. Percentages of bootstrap values are shown at the branch node. A subscript “A” was assigned arbitrarily to indicate the cluster obtained with Asian DS-1-like G1P[8], equine-like G3P[8] DS-1-like strains, and Brazilian DS-1-like G1P[8] strains.

Phylogenetic analysis using a DS-1-like backbone together with representative G2P[4], G8P[6], G9P[4], G12P[6], G6P[6] and G12P[4] RVAs from all over the world indicated that most of the Brazilian DS-1-like G1P[8] RVA genome segments were occupied a single lineage: VP1, VP3, VP6, NSP2 and NSP3 in Lineage V; and VP2, NSP1 and NSP5 in Linage IVa. This lineage constellation was the same as in the Asian DS-1-like G1P[8] strains that emerged in emerged in Japan, Thailand, Philippines and Vietnam between 2012 and 2014^10–13,15^, as well as in the contemporary equine-like G3P[8] DS-1-like strains detected after 2013 in Australia, Japan, Spain, United States, Thailand and Hungary^6–8,34^. The branches are also supported with high bootstrap values, and a subscript “A” was assigned arbitrarily. RVA/Human-wt/MWI/BID2BS/2013/G1P[8] strain, detected in Africa^14^, did not cluster into subscript “A” with strains from Australia, USA, Canada, and Europe, in addition to strains from Brazil and Asia, even when showing high mean identities ranging from 91.3–98.0% (Supplement 6). The DS-1-like genes of the G1P[8] reassortants detected in the present study distantly clustered with those of DS-1-like backbone strains that circulated earlier in Brazil, therefore, they did not emerge through reassortment among local strains (Fig. 1A–C and E–I).

Considering NSP4 gene analysis in particular, Brazilian IAL-R3122/2013, IAL-R3123, IAL-R3165/2013 and IAL-R3172/2013 strains exhibited close genetic relationship to those of Japan (HC12016), Philippines (TGO12-004), Thailand (PCB-180) and Vietnam (SP026) DS-1-like G1P[8] strains (90.8–98.5% nt; Lineage VI) in a common branch with equine like G3P[8] DS-1-like strains from Spain (SS98244047) and Japan (IS1078) (97.4–97.8% nt); however, away from the cluster comprising the DS-1-like G1P[8] strains also detected in Japan (KN041 and OH3625) and Malawi (BID2BS) (84.8–91.4% nt; Lineage VII) (Fig. 1D, Supplement 6).

Based on the VP4 phylogenetic tree, the Brazilian DS-1-like G1P[8] detected here were grouped into the Lineage III, as expected (Fig. 1K). After 2003, virtually all globally circulating P[8] strains belonged to Lineage III. Brazilian DS-1-like G1P[8] Brazilian IAL-R3122/2013, IAL-R3123, IAL-R3165/2013 and IAL-R3172/2013VP4 gene segments are similar to all DS-1-like G1P[8] strains emerging worldwide (94.4–99.3% nt) and to those equine like G3P[8] DS-1-like strains (98.3–98.5% nt) (Supplement 6). On the other hand, for the VP7 gene, the Brazilian DS-1-like G1P[8] strains were grouped into Lineage I, apart from the Rotarix^TM^ and Rotateq^TM^ vaccine strains (Lineage II and III, respectively). Strains belonging to Lineage I were largely detected after 1997^29^, and the nt identities range from 90.5 to 98.96% compared to other DS-1-like G1P[8] strains. A key observation was extracted from the phylogenetic analysis of the VP7 gene. The former reported African BID2BS DS-1-like G1P[8] strain^14^ could not be classified in any lineage proposed by Arista *et al*.^31^ and exhibited low nt identity related to DS-1-like G1P[8] strains (85.1%) (Fig. 1J, Supplement 6).

## Discussion

This study documents the whole genome characterization of DS-1-like G1P[8] strains detected for the first time in the Americas. As universal RVA vaccination continues to increase worldwide, global surveillance of RVA has become an important focus of vaccination programs and is vital for monitoring the emergence of new strains^6,7,14,28,35^. Brazil has a high national RVA vaccine coverage (~92.0%) (http://tabnet.datasus.gov.br). Even after the introduction of Rotarix^TM^, the G1P[8] strain continued to circulate over time at very low levels^28^, similar to the frequency observed in the present study, suggesting that the G1P[8] detection was not linked to a gap in vaccination.

Atypical reassortant DS-1-like G1P[8] strains emerged during the 2012/2013 season in Southeast Asia^10–13,15^ and were also recently reported in Malawi, Africa^14^. The Jere *et al*.^14^ study raises the question of if the DS-1-like G1P[8] strains emergences could be linked to RVA vaccination introduction. The detection of African DS-1-like G1P[8] strains seems to coincided with widespread use of a G1P[8] Rotarix™ RVA vaccine in Malawi after 2013 and Southeast Asian countries are known to possess limited RVA vaccine use. Rotarix^TM^ was included in the Brazilian Immunization Program in 2006; and during these ~10 years of post-vaccine surveillance, no atypical DS-1-like G1P[8] strain was reported, except for these particular 13 samples identified in 2013. Moreover, the genetic analysis indicated that the DS-1-like G1P[8] strains detected in Brazil were not derived from a reassortant with vaccine VP4 and VP7 strains, since the outer-capsid genes did not belong to the same Rotarix^TM^ vaccine lineages and low nucleotide identities were observed. Therefore, our data suggested that the emergence of DS-1-like G1P[8] is unlikely to be associated with the RVA vaccine introduction in Brazil.

Atypical DS-1-like G1P[8] strains from Brazil and Asia exhibited similar phylogenetic clustering patterns in all 11 genome segments, including G and P genes, and grouped away from the Malawian DS-1-like G1P[8] reassortant. In particular, the NSP4 E2 genotype identified in the DS-1-like G1P[8] Malawian strain (BID2BS) and two Japanese strains (KN041 and OH3625) detected in 2012 belong to Lineage VII, while NSP4 E2 genotype recognized in DS-1-like G1P[8] strains detected in Brazil belong to Lineage VI and grouped together with the Asian DS-1-like G1P[8] strains detected in Japan also in 2012, along with strains from Philippines and Thailand. Therefore, globally co-circulating pool of different DS-1-like G1P[8] strains could be suggested.

Monophyletic clusters between non-G and non-P genes of Brazilian DS-1-like G1P[8] strains with other Brazilian DS-1-like strains was not observed. The VP7 and VP4 genes of strains RVA/Human-wt/BRA/IAL-R3122/2013/G1P[8], RVA/Human-wt/BRA/IAL-R3123/2013/G1P[8], RVA/Human-wt/BRA/IAL-R3165/2013/G1P[8] and RVA/Human-wt/BRA/IAL-R3172/2013/G1P[8] were distantly related to those of Wa-like G1P[8] strains isolated in Brazil and America in the same period. The Brazilian DS-1-like G1P[8] strains also exhibited limited circulation, considering time (only in 2013) and space (only in São Paulo and Goiás states), compared to their widespread circulation in Asian countries^12,16^. These data together indicated that the atypical Brazilian DS-1-like G1P[8] strains are likely to be imported from Asia. Currently, Brazil is the country with the largest Japanese population outside Japan; conversely, the Brazilian community in Japan is the third largest community of foreign workers that reside in the country^36^. In addition, São Paulo State holds the largest Chinese colony of the country^37^. The idea that this migration flow may lead to an exchange of RVA strains is one that can be speculated upon; however, additional in-depth analysis of full length of DS-1-like G1P[8] gene sequences collected worldwide is required to fully validate this hypothesis.

The emergence of equine DS-1-like G3P[8] strains in Australia^7^, Thailand^11^, Spain^6^, and Brazil^9^ merits attention because (i) these strains also possessed the DS-1-like VP1-4, VP6, and NSP1-5/6 protein genes and (ii) the Brazilian DS-1-like G1P[8] strains clustered together with these contemporary equine-like G3P[8] DS-1-like strains. It has been suggested that the G3P[8] double-reassortant strains were originated from DS-1-like G1P[8] strains^11,12^. The 10 characterized genes (except VP7 gene) of the Brazilian DS-1-like G1P[8] strains were also closely related with the recently emerged equine-like DS-1-like G3P[8] strains. Thus, the DS-1-like G1P[8] and equine-like DS-1-like G3P[8] strains may actually have a common origin. The rapidly increasing detection of novel emerging RVA strains, in association with the genetic heterogeneity, raises intriguing questions about RVA evolution^38^. Further definition of the cut off value between lineages and sub-lineages for each gene segment, as well as an in-depth phylodynamic analysis is required to shed some light on better understanding the complex dynamics of these emerging RVA strains.

This is the first full genome characterization of DS-1-like G1P[8] strains that have emerged in the Americas. Its detection, together with the Jere *et al*.^14^ report, may imply the ongoing spread of these unusual RVA strains outside of Asia. PCR-based genotyping allied with PAGE analysis could be easily applied to detect atypical DS-1-like G1P[8] strains, although whole genome-based analysis are still essential. Global RVA strain collection is imperative in order to determine the exact origin(s) of the DS-1-like G1P[8] strains. In addition, an intensive, distinct surveillance system, focused on determining if the available RVA vaccines are still effective against unusual DS-1-like G1P[8] strains, as well as investigating whether these recently emerged G1P[8] double-gene reassortant strains cause more-severe disease^12^, must be developed.

## Supplementary information

Supplementary info
