# Supplementary material for: First Detection of DS-1-like G1P[8] Double-gene Reassortant Rotavirus Strains on The American Continent, Brazil, 2013
Source: Sci Rep. 2019 Feb 18;9:2210. doi: 10.1038/s41598-019-38703-7 (PMC6379365; doi:10.1038/s41598-019-38703-7)
Supplement: Supplementary file 1 — Supplementary info [file 41598_2019_38703_MOESM1_ESM.pdf]

FIRST DETECTION OF DS-1-LIKE G1P[8] DOUBLE-GENE REASSORTANT ROTAVIRUS STRAINS ON THE AMERICAN CONTINENT, BRAZIL, 2013

Adriana Luchs[1,*], Antonio Charlys da Costa[2,3], Audrey Cilli[1], Shirley Cavalcante Vasconcelos Komninakis[4,5], Rita de Cássia Compagnoli Carmona[1], Simone Guadagnucci Morillo[1], Ester Cerdeira Sabino[2,3], Maria do Carmo Sampaio Tavares Timenetsky[1,*]

[1]Enteric Disease Laboratory, Virology Center, Adolfo Lutz Institute, São Paulo, Brazil

[2]LIM/46 - Laboratory of Medical Parasitology, Department of Infectious and Parasitic Diseases, College of Medicine, University of São Paulo, São Paulo, Brazil

[3]Institute of Tropical Medicine, University of São Paulo, São Paulo, Brazil

[4]Postgraduate Program in Health Science, Faculty of Medicine of ABC, Santo André, Brazil

[5]Retrovirology Laboratory, Federal University of São Paulo, São Paulo, São Paulo, Brazil

*Corresponding author: Adriana Luchs, Maria do Carmo Sampaio Tavares Timenetsky

Instituto Adolfo Lutz, Centro de Virologia, Núcleo de Doenças Entéricas.

Address: Av. Dr Arnaldo, nº 355, São Paulo, SP, Brasil, 01246-902

Fax: 55-11-3088 3753; phone: 55-11-3068 2909

e-mail: **driluchs@gmail.com**; **timenetsky.m@gmail.com**

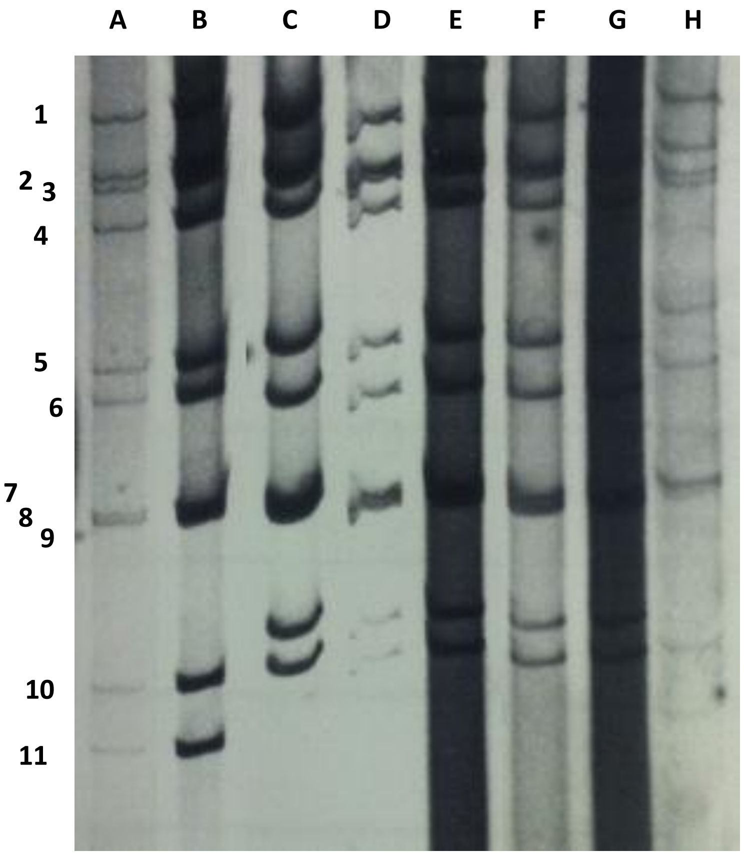

Supplement 1. Poliacrylamide gel electropheresis of G1P[8] RVA strains identified in Brazil, 2013-2017. Lines A and B: Wa-like G1P[8] strains long electropherotype; Lines C to G: DS-1-like G1P[8] strains short electropherotype; Line H: SA11 positive control. Numbers are indicating the eleven RVA dsRNA segments.

Supplement 2. Socio-demographic data of Brazilian patients with Wa-like G1P[8] and DS-1-like G1P[8] RVA infection, 2013-2017.

Age	Sex	City	State	Month	Year	Electrophoresis profile	Genotype	RVA Vaccine	Strain
9 months	M	São Paulo	SP	March	2013	Long	Wa-like G1P[8]	Yes[ǂ]	---
8 months	M	São Paulo	SP	August	2013	Long	Wa-like G1P[8]	*	---
2 years	F	São Paulo	SP	August	2013	Short	DS-1-like G1P[8]	No	---
3 years	F	São Paulo	SP	September	2013	Short	DS-1-like G1P[8]	No	---
1 year	M	Mogi das Cruzes	SP	September	2013	Short	DS-1-like G1P[8]	*	IAL-R3122
5 years	F	Mogi das Cruzes	SP	September	2013	Short	DS-1-like G1P[8]	*	IAL-R3123
3 years	F	Mogi das Cruzes	SP	September	2013	Short	DS-1-like G1P[8]	*	---
5 years	F	Guarulhos	SP	September	2013	Long	Wa-like G1P[8]	*	---
77 years	F	Guarulhos	SP	September	2013	Long	Wa-like G1P[8]	No	---
6 months	F	São Paulo	SP	September	2013	Long	Wa-like G1P[8]	*	---
1 years	M	São Paulo	SP	September	2013	Long	Wa-like G1P[8]	Yes[ǂ]	---
1 years	M	São Paulo	SP	September	2013	Short	DS-1-like G1P[8]	*	IAL-R3165

1 years	F	São Paulo	SP	September	2013	Short	DS-1-like G1P[8]	Yes[†]	---
48 years	F	Osasco	SP	September	2013	Short	DS-1-like G1P[8]	No	---
8 years	F	São Luis de Belos Montes	GO	September	2013	Short	DS-1-like G1P[8]	No	IAL-R3172
3 years	F	São Paulo	SP	September	2013	Short	DS-1-like G1P[8]	Yes[†]	---
1 year	M	Guarulhos	SP	October	2013	Short	DS-1-like G1P[8]	Yes[†]	---
7 years	M	Guarulhos	SP	October	2013	Short	DS-1-like G1P[8]	*	---
12 years	M	São Luis de Belos Montes	GO	October	2013	Short	DS-1-like G1P[8]	No	---
3 years	F	Matelandia	PR	October	2013	Long	Wa-like G1P[8]	*	---
2 years	M	Matelandia	PR	October	2013	Long	Wa-like G1P[8]	*	---
2 years	M	Matelandia	PR	October	2013	Long	Wa-like G1P[8]	*	---
2 years	F	Matelandia	PR	October	2013	Long	Wa-like G1P[8]	*	---
3 years	M	Matelandia	PR	October	2013	Long	Wa-like G1P[8]	*	---
1 year	M	Matelandia	PR	October	2013	Long	Wa-like G1P[8]	*	---
10 years	M	Matelandia	PR	October	2013	Long	Wa-like G1P[8]	*	---
---	F	Goiânia	GO	November	2013	Long	Wa-like G1P[8]	*	---

2 years	M	Pontal do Paraná	PR	February	2014	Long	Wa-like G1P[8]	*	---
33 years	M	Goiânia	GO	April	2014	Long	Wa-like G1P[8]	*	---
2 months	M	São Paulo	SP	July	2015	Long	Wa-like G1P[8]	Yes[ǂ]	---

State of São Paulo (SP), State of Goiás (GO), State of Paraná (PR)
*Not known
[ǂ]Number of doses not informed

Supplement 3. Sequence data for the 11 genome segments of four DS-1-like G1P[8] rotaviruses

Strain	Sequence reads	Genome segments								
		**VP1**	**VP2**	**VP3**	**VP4**	**VP6**	**VP7**	**NSP1**	**NSP2**	**NSP3**
RVA/Human-wt/BRA/IAL-R3122/2013/G1P[8]	Length	3302	2684	2591	2359	1356	1062	1566	1059	1066
	Total reads for each segments	97x	49x	48x	43x	239x	37x	29x	44x	18x
RVA/Human-wt/BRA/IAL-R3123/2013/G1P[8]	Length	3302	2684	2591	2359	1356	1062	1566	1059	1066
	Total reads for each segments	2130x	1071x	846x	748x	1520x	574x	303x	472x	220x
RVA/Human-wt/BRA/IAL-R3165/2013/G1P[8]	Length	3302	2684	2591	2359	1356	1062	1566	1059	1066
	Total reads for each segments	61x	18x	14x	18x	58x	12x	25x	16x	10x
RVA/Human-wt/BRA/IAL-R3172/2013/G1P[8]	Length	3302	2684	2591	2359	1356	1062	1566	1059	1066
	Total reads for each segments	287x	177x	145x	145x	473x	136x	55x	144x	48x

Supplement 4. Raw data of the sequence of four Brazilian DS-1-like G1P[8] rotaviruses.

Sample	Total reads >Q30	% Human	% Bacteria	% Fungus	Other viroses*
RVA/Human-wt/BRA/IAL-R3122/2013/G1P[8]	18100	0	1	0	9
RVA/Human-wt/BRA/IAL-R3123/2013/G1P[8]	46782	0	0	0	19
RVA/Human-wt/BRA/IAL-R3165/2013/G1P[8]	22382	0	0	0	8
RVA/Human-wt/BRA/IAL-R3172/2013/G1P[8]	22818	0	0	0	22

*Other viruses identified in the samples were not evaluated.

Supplement 5. The genotype constellation of four sequenced Brazilian DS-1-like G1P[8] as well as the reference strains used in the phylogenetic analysis.

	Name of strain	Country of isolation	Year of isolation	VP7	VP4	VP6	VP1	VP2	VP3	NSP1	NSP2	NSP3	NSP4
1	IAL-R3122	Brazil	2013	G1	P[8]	I2	R2	C2	M2	A2	N2	T2	E2
2	IAL-R3123	Brazil	2013	G1	P[8]	I2	R2	C2	M2	A2	N2	T2	E2
3	IAL-R3165	Brazil	2013	G1	P[8]	I2	R2	C2	M2	A2	N2	T2	E2
4	IAL-R3172	Brazil	2013	G1	P[8]	I2	R2	C2	M2	A2	N2	T2	E2
5	SKT-109	Thailand	2013	G1	P[8]	I2	R2	C2	M2	A2	N2	T2	E2
6	BID2BS	Malawi	2013	G1	P[8]	I2	R2	C2	M2	A2	N2	T2	E2
7	HC12016	Japan	2012	G1	P[8]	I2	R2	C2	M2	A2	N2	T2	E2
8	PCB-180	Thailand	2013	G1	P[8]	I2	R2	C2	M2	A2	N2	T2	E2
9	To14-41	Japan	2014	G1	P[8]	I2	R2	C2	M2	A2	N2	T2	E2
10	SP026	Vietnam	2012	G1	P[8]	I2	R2	C2	M2	A2	N2	T2	E2
11	TGO12-004	Philippines	2012	G1	P[8]	I2	R2	C2	M2	A2	N2	T2	E2
12	KN041	Japan	2012	G1	P[8]	I2	R2	C2	M2	A2	N2	T2	E2
13	TGO12-045	Philippines	2012	G1	P[8]	I2	R2	C2	M2	A2	N2	T2	E2
14	OH3625	Japan	2012	G1	P[8]	I2	Rx	Cx	Mx	Ax	Nx	Tx	E2
15	Dhaka16	Bangladesh	2003	G1	P[8]	I1	R1	C1	M1	A1	N1	T1	E1
16	Rotarix	United States of America	1988	G1	P[8]	I1	R1	C1	M1	A1	N1	T1	E1
17	Rotateq	United States of America	1992	G1	P[5]	I2	R2	C2	M1	A3	N2	T6	E2
18	BE00094	Belgium	2009	G1	P[8]	I1	R1	C1	M1	A1	N1	T1	E1
19	B74-02	Belgium	2002	G1	P[8]	I1	R1	C1	M1	A1	N1	T1	E1
20	R321-2004	Brazil	2004	G1	P[8]	I1	R1	C1	M1	A1	N1	T1	E1
21	2007719635	United States of America	2007	G1	P[8]	I1	R1	C1	M1	A1	N1	T1	E1
22	QUI-150-F1	Brazil	2010	G1	P[8]	I1	R1	C1	M1	A1	N1	T1	E1
23	CU957-KK/11	Thailand	2011	G1	P[8]	I1	R1	C1	M1	A1	N1	T1	E1
24	D	United States of America	1974	G1	P[8]	I1	R1	C1	M1	A1	N1	T1	E1
25	9J	Nicaragua	2010	G1	P[8]	I1	R1	C1	M1	A1	N2	T1	E1
26	CK00083	Australia	2008	G1	P[8]	Ix	Rx	Cx	Mx	Ax	Nx	Tx	Ex
27	PA258	Italy	1997	G1	P[8]	Ix	Rx	Cx	Mx	Ax	Nx	Tx	Ex
28	Mvd9806	Uruguay	1996	G1	P[8]	Ix	Rx	Cx	Mx	Ax	Nx	Tx	Ex
29	PA78/89	Italy	1989	G1	P[8]	Ix	Rx	Cx	Mx	Ax	Nx	Tx	Ex
30	PA10/90	Italy	1990	G1	P[8]	Ix	Rx	Cx	Mx	Ax	Nx	Tx	Ex
31	PA33/99	Italy	1999	G1	P[8]	Ix	Rx	Cx	Mx	Ax	Nx	Tx	Ex
32	Mvd9606	Uruguay	1996	G1	P[8]	Ix	Rx	Cx	Mx	Ax	Nx	Tx	Ex
33	Py03IPN144	Paraguay	2002-2005	G1	P[8]	Ix	Rx	Cx	Mx	Ax	Nx	Tx	Ex
34	Brz-6	Brazil	1995	G1	P[8]	Ix	Rx	Cx	Mx	Ax	Nx	Tx	Ex
35	87Y1397	Japan	xxxx	G1	P[8]	Ix	Rx	Cx	Mx	Ax	Nx	Tx	Ex
36	k54	United States of America	xxxx	G1	P[x]	Ix	Rx	Cx	Mx	Ax	Nx	Tx	Ex
37	Kor-64	South Korea	1988	G1	P[x]	Ix	Rx	Cx	Mx	Ax	Nx	Tx	Ex
38	Isr-56	Israel	1995	G1	P[x]	Ix	Rx	Cx	Mx	Ax	Nx	Tx	Ex
39	AU19	Japan	xxxx	G1	P[x]	Ix	Rx	Cx	Mx	Ax	Nx	Tx	Ex
40	C60	Venezuela	xxxx	G1	P[x]	Ix	Rx	Cx	Mx	Ax	Nx	Tx	Ex
41	T449	Unknown	xxxx	G1	P[x]	Ix	Rx	Cx	Mx	Ax	Nx	Tx	Ex
42	R70	Brazil	1997	G1	P[9]	I1	R1	C1	M3	A1	N1	T2	E1
43	SS98244047	Spain	2015	G3	P[8]	I2	R2	C2	M2	A2	N2	T2	E2
44	SKT-239	Thailand	2013	G3	P[8]	I2	R2	C2	M2	A2	N2	T2	E2
45	3000390639	United States of America	2015	G3	P[8]	I2	R2	C2	M2	A2	N2	T2	E2
46	ERN8263	Hungary	2015	G3	P[8]	I2	R2	C2	M2	A2	N2	T2	E2
47	WAPC2016	Australia	2014	G3	P[8]	I2	R2	C2	M2	A2	N2	T2	E2
48	WAPC1740	Australia	2013	G3	P[8]	I2	R2	C2	M2	A2	N2	T2	E2
49	IS1078	Japan	2015	G3	P[8]	I2	R2	C2	M2	A2	N2	T2	E2
50	ITO	Japan	1981	G3	P[8]	Ix	Rx	Cx	Mx	Ax	Nx	Tx	Ex
51	BE87	Belgium	2011	G2	P[4]	I2	R2	C2	M2	A2	N2	T2	E2
52	07Y003	Japan	2007	G2	P[4]	I2	R2	C2	M2	A2	N2	T2	E2
53	PA108	Italy	2007	G2	P[4]	I2	R2	C2	M2	A2	N2	T2	E2
54	J306	Bangladesh	2010	G2	P[4]	I2	R2	C2	M2	A2	N2	T2	E2

55	J263	Bangladesh	2010	G2	P[4]	I2	R2	C2	M2	A2	N2	T2	E2
56	LB2772	United States of America	2005	G2	P[4]	I2	R2	C2	M2	A2	N2	T2	E2
57	LB2764	United States of America	2005	G2	P[4]	I2	R2	C2	M2	A2	N2	T2	E2
58	M334	Bangladesh	2013	G2	P[4]	I2	R2	C2	M2	A2	N2	T2	E2
59	MMC6	Bangladesh	2005	G2	P[4]	I2	R2	C2	M2	A2	N2	T2	E2
60	TB-Chen	China	1996	G2	P[4]	I2	R2	C2	M2	A2	N2	T2	E2
61	86Y1329	Japan	1986	G2	P[4]	I2	R2	C2	M2	A2	N2	T2	E2
62	KUN	Japan	1980	G2	P[4]	I2	R2	C2	M2	A2	N2	T2	E2
63	D205	Kenya	1989	G2	P[4]	I2	R2	C2	M2	A2	N2	T2	E2
64	89Y1520	Japan	1989	G2	P[4]	I2	R2	C2	M2	A2	N2	T2	E2
65	83A001	Japan	1983	G2	P[4]	I2	R2	C2	M2	A2	N2	T2	E2
66	SE19300	Brazil	2010	G2	P[4]	I2	R2	C2	M2	A2	N2	T2	E2
67	MS11142	Brazil	2005	G2	P[4]	I2	R2	C2	M2	A2	N2	T2	E2
68	QUI-60-F1	Brazil	2008	G2	P[4]	I2	R2	C2	M2	A2	N2	T2	E2
69	ES16238	Brazil	2009	G2	P[4]	I2	R2	C2	M2	A2	N2	T2	E2
70	RJ12225	Brazil	2006	G2	P[4]	I2	R2	C2	M2	A2	N2	T2	E2
71	MA14286	Brazil	2007	G2	P[4]	I2	R2	C2	M2	A2	N2	T2	E2
72	SC19868	Brazil	2011	G2	P[4]	I2	R2	C2	M2	A2	N2	T2	E2
73	CK20001	Australia	1977	G2	P[4]	I2	R2	C2	M2	A2	N2	T2	E2
74	CK20033	Australia	2006	G2	P[4]	I2	R2	C2	M2	A2	N2	T2	E2
75	CK20047	Australia	2011	G2	P[4]	I2	R2	C2	M2	A2	N2	T2	E2
76	B110119	Japan	2011	G2	P[4]	I2	R2	C2	M2	A2	N2	T2	E2
77	2007769964	United States of America	2007	G2	P[4]	I2	R2	C2	M2	A2	N2	T2	E2
78	PAI11	Italy	1996	G2	P[4]	I2	R2	C2	M2	A2	N2	T2	E2
79	B110056	Japan	2011	G2	P[4]	I2	R2	C2	M2	A2	N2	T2	E2
80	83A001	Japan	1983	G2	P[4]	I2	R2	C2	M2	A2	N2	T2	E2
81	MMC88	Bangladesh	2005	G2	P[4]	I2	R2	C2	M2	A2	N2	T2	E2
82	RT128-07	Canada	2008	G2	P[4]	I2	R2	C2	M2	A2	N2	T2	E2
83	Ghan-148	Ghana	2007	G2	P[4]	I2	R2	C2	M2	A2	N2	T2	E2
84	J253	Bangladesh	2010	G2	P[4]	I2	R2	C2	M2	A2	N2	T2	E2
85	V233	Australia	199	G2	P[4]	I2	R2	C2	M2	A2	N2	T2	E2
86	116E3D	India	1999	G2	P[4]	I2	R2	C2	M2	A2	N2	T2	E2
87	J303	Bangladesh	2010	G2	P[4]	I2	R2	C2	M2	A2	N2	T2	E2
88	90A009	Japan	1990	G2	P[4]	I2	R2	C2	M2	A2	N1	T2	E2
89	90H094	Japan	1990	G2	P[4]	I2	R2	C2	M2	A2	N1	T2	E2
90	AK26	Kenya	1982	G2	P[4]	I2	R2	C2	M2	A2	N1	T2	E2
91	CU616-TK/09	Thailand	2009	G12	P[8]	Ix	Rx	Cx	Mx	Ax	Nx	Tx	Ex
92	RJ12419	Brazil	2006	G12	P[8]	I1	R1	C1	M1	A1	N1	T1	E1
93	QUI-154-F1	Brazil	2010	G12	P[6]	I2	R2	C2	M2	A2	N2	T2	E2
94	RV161	Bangladesh	2010	G12	P[6]	I2	R2	C2	M2	A2	N2	T2	E1
95	L26	Phillippines	1987	G12	P[4]	I2	R2	C2	M1	A2	N1	T2	E2
96	OP530	Malawi	1999	G4	P[8]	Ix	Rx	Cx	Mx	Ax	Nx	Tx	Ex
97	MW670	Malawi	1999	G4	P[8]	Ix	Rx	Cx	Mx	Ax	Nx	Tx	Ex
98	B1711	Belgium	2002	G6	P[6]	I2	R2	C2	M2	A2	N2	T2	E2
99	469	Paraguay	2000	G9	P[8]	I1	R1	C1	M1	A1	N1	T1	E1
100	F45	Japan	1987	G9	P[8]	Ix	Rx	Cx	Mx	Ax	Nx	Tx	Ex
101	RV11	India	2011	G9	P[4]	I2	R2	C2	M2	A2	N2	T2	E6
102	DRC86	Democratic Republic of the Congo	2003	G8	P[6]	I2	R2	C2	M2	A2	N2	T2	E2

DS-1-like G1P[8] strains determined in this study: name in red

Supplement 6. Gene segments' nucleic acid identity of selected DS-1-like G1P[8] strains. Nucleic acid sequences of all eleven gene segments from Brazilian strains IAL-R3122 (A), IAL-R3123 (B), IAL-R3165 (C), and IAL-R3172 (D) were compared with others DS-1-like G1P[8] strains. For comparison, strains from GenBank were selected as indicated in Figure 1. Identities within the gene segments ORF are given in percentage. Nucleic acid identities below 90% are highlighted in bold face.

(A)

	Nucleotide identity (%)										
**IAL-R3122**	**VP7**	**VP4**	**VP1**	**VP2**	**VP3**	**VP6**	**NSP1**	**NSP2**	**NSP3**	**NSP4**	**NSP5**
RVA/Human-wt/BRA/IAL-R3123/2013/G1P[8]	100	99.9	99.8	99.9	99.9	99.8	100	99.9	99.9	99.8	99.8
RVA/Human-wt/BRA/IAL-R3165/2013/G1P[8]	100	99.8	99.4	99.8	99.9	100	99.8	99.9	99.6	99.8	99.8
RVA/Human-wt/BRA/IAL-R3172/2013/G1P[8]	100	99.8	99.6	99.7	99.9	99.8	99.8	99.8	99.7	100	99.7
RVA/Human-wt/MWI/BID2BS/2013/G1P[8]	**85.1**	94.4	94.0	97.2	95.9	98.0	97.1	97.1*	97.2	91.3	97.7
RVA/Human-wt/JPN/OH3625/2012/G1P[8]	-	-	-	-	-	-	-	-	-	**86.2**	-
RVA/Human-wt/JPN/KN041/2012/G1P[8]	-	-	-	-	-	-	-	-	-	**84.8**	-
RVA/Human-wt/JPN/HC12016/2012/G1P[8]	93.5*	99.1	99.0	98.3*	97.9*	93.8*	-	91.7*	92.7*	90.8*	**89.0***
RVA/Human-wt/THA/PCB-180/2013/G1P[8]	98.3	97.7	99.4	99.1	98.9	98.8	-	98.0	99.1	97.2	97.0*
RVA/Human-wt/PHI/TGO12-004/2012/G1P[8]	98.6	-	-	-	-	97.9	-	97.3	99.4	98.4	-
RVA/Human-wt/PHI/TGO12-045/2012/G1P[8]	-	98.6	-	99.6	98.9	-	99.6	-	-	-	99.3
RVA/Human-wt/THA/SKT-109/2013/G1P[8]	-	-	-	-	-	-	99.6	-	-	-	-
RVA/Human-wt/JPN/To14-41/2014/G1P[8]	-	-	-	-	-	-	97.8*	-	-	-	-
RVA/Human-wt/VNM/SP026/2012/G1P[8]	90.5*	97.7	98.1	97.4*	96.6*	96.1*	96.4*	94.1*	94.9	92.2*	94.7*
RVA/Human-wt/BGD/Dhaka16/2003/G1P[8]	96.3	97.4	-	-	-	-	-	-	-	-	-
RVA/Vaccine/USA/Rotarix-AROLA490AB/1988/G1P[8]	**89.7***	**89.3**	-	-	-	-	-	-	-	-	-
RVA/Vaccine/USA/RotaTeq-WI79-9/1992/G1P7[5]	91.6	91.6	-	-	-	-	-	-	-	-	-
RVA/Human-wt/BEL/B74-02/2002/G1P[8]	**89.1***	-	-	-	-	-	-	-	-	-	-
RVA/Human-wt/USA/2007719635/2007/G1P[8]	94.7*	-	-	-	-	-	-	-	-	-	-
RVA/Human-wt/USA/CU957-KK/11/2011/G1P[8]	**87.1***	-	-	-	-	-	-	-	-	-	-
RVA/Human-tc/USA/D/1974/G1P[8]	**84.5***	-	-	-	-	-	-	-	-	-	-
RVA/Human-wt/USA/Wa/1974/G1P[8]	-	**89.6**	-	-	-	-	-	-	-	-	-
RVA/Human-wt/BEL/BE00094/2009/G1P[8]	-	95.3	-	-	-	-	-	-	-	-	-
RVA/Human-tc/USA/K54/xxxx/G1P[x]	**84.9***	-	-	-	-	-	-	-	-	-	-
RVA/Human-wt/JPN/AU19/xxxx/G1P[x]	**78.8***	-	-	-	-	-	-	-	-	-	-
RVA/Bovine-wt/XXX/T449/xxxx/G1P[x]	**85.7**	-	-	-	-	-	-	-	-	-	-
RVA/Human-wt/BRA/QUI-150-F1/2010/G1P[8]	**88.2***	-	-	-	-	-	-	-	-	-	-
RVA/Human-wt/BRA/R321-2004/2004/G1P[x]	91.0	-	-	-	-	-	-	-	-	-	-
RVA/Human-tc/BRA/R70/1997/G1P[9]	91.0*	-	-	-	-	-	-	-	-	-	-
RVA/Human-wt/PAR/Py03IPN144/2002-2005/G1P[8]	**85.7***	-	-	-	-	-	-	-	-	-	-
RVA/Pig-tc/VEN/C60/xxxx/G1P[x]	**84.5**	-	-	-	-	-	-	-	-	-	-
RVA/Human-wt/ISR/Isr-56/1995/G1P[x]	**85.1***	-	-	-	-	-	-	-	-	-	-
RVA/Human-wt/BRA/Brz-6/1995/G1P[x]	**84.1***	-	-	-	-	-	-	-	-	-	-
RVA/Human-wt/JPN/87Y1397/xxxx/G1P[8]	92.9	-	-	-	-	-	-	-	-	-	-
RVA/Human-wt/KOR/Kor-64/1988/G1P[x]	**85.4***	-	-	-	-	-	-	-	-	-	-
RVA/Human-wt/ITA/PA10/90/1990/G1P[8]	**83.5***	-	-	-	-	-	-	-	-	-	-
RVA/Human-wt/ITA/PA78/89/1989/G1P[8]	**84.0***	-	-	-	-	-	-	-	-	-	-
RVA/Human-wt/URY/Mvd9606/1996/G1P[8]	94.1	-	-	-	-	-	-	-	-	-	-
RVA/Human-wt/ITA/PA33/99/1999/G1P[8]	**83.3***	-	-	-	-	-	-	-	-	-	-
RVA/Human-wt/NCA/9J/2010/G1P[8]	**88.4***	97.1	-	-	-	-	-	-	-	-	-
RVA/Human-wt/AUS/CK00083/2008/G1P[8]	92.5*	-	-	-	-	-	-	-	-	-	-
RVA/Human-wt/URY/Mvd9806/1996/G1P[8]	97.1	-	-	-	-	-	-	-	-	-	-
RVA/Human-wt/ITA/PA258/97/1997/G1P[8]	**82.7***	-	-	-	-	-	-	-	-	-	-
RVA/Human-wt/AUS/WAPC1740/2013/G3P[8]	-	-	-	-	-	-	99.6	99.7	-	-	-
RVA/Human-wt/AUS/WAPC2016/2014/G3P[8]	-	-	-	99.3	98.7	99.5	-	-	-	-	99.5
RVA/Human-wt/ESP/SS98244047/2015/G3P[8]	-	-	-	-	-	-	-	99.4	99.3	97.7	99.1
RVA/Human-wt/JPN/IS1078/2015/G3P[8]	-	-	-	-	-	99.1	99.3	-	99.5	97.4	-
RVA/Human-wt/USA/3000390639/2015/G3P[8]	-	-	98.6	-	-	-	-	-	-	-	-
RVA/Human-wt/HUN/ERN8263/2015/G3P[8]	-	-	98.8	-	-	-	-	-	-	-	-
RVA/Human-wt/THA/SKT-289/2013/G3P[8]	-	98.3	-	99.2	99.2	-	-	-	-	-	-
RVA/Human-wt/USA/DC23/1976/G3P[8]	-	**89.6***	-	-	-	-	-	-	-	-	-
RVA/Human-wt/AUS/D388/2013/G3P[8]	-	98.8	-	-	-	-	-	-	-	-	-
RVA/Human-wt/JPN/ITO/1981/G3P[8]	-	**88.7**	-	-	-	-	-	-	-	-	-
RVA/Human-wt/BEL/BE87/2011/G2P[4]	-	-	-	-	-	-	91.6*	**87.4***	-	**49.9***	**72.5***
RVA/Human-wt/ITA/PA108/2007/G2P[4]	-	-	-	-	95.9	98.6	97.8*	-	-	-	-
RVA/Human-wt/JPN/07Y003/2007/G2P[4]	-	-	-	-	-	-	90.6*	-	-	-	-
RVA/Human-wt/BGN/J306/2010/G2P[4]	-	-	-	-	-	-	98.6	-	-	-	-
RVA/Human-wt/BRA/QUI-60-F1/2008/G2P[4]	-	-	-	-	-	-	96.1*	94.7*	91.2*	94.6	96.3
RVA/Human-wt/BRA/SE19300/2010/G2P[4]	-	-	-	-	-	-	96.1*	90.8*	91.0*	**79.6***	90.1*

RVA/Human-wt/BRA/MS11142/2005/G2P[4]	-	-	97.3	-	-	-	96.6*	91.4*	91.9*	**78.4***	91.6*
RVA/Human-wt/JPN/90A009/1990/G2P[4]	-	-	-	-	-	-	-	-	**89.4***	**82.6***	-
RVA/Human-wt/JPN/90H094/1990/G2P[4]	-	-	-	92.9*	-	-	-	-	-	-	-
RVA/Human-wt/JPN/83A001/1983/G2P[4]	-	-	**88.4***	**89.5***	91.1*	**81.0***	**84.4***	**76.6**	**86.3***	**77.6***	**81.8***
RVA/Human-wt/JPN/89Y1520/1989/G2P[4]	-	-	**87.0***	-	91.2*	**80.6***	**85.5***	-	**87.5***	**84.2***	**85.6***
RVA/Human-tc/KEN/D205/1989/G2P[4]	-	-	**86.3**	93.6	-	-	95.0	96.1	96.4	90.1	93.6
RVA/Human-wt/KEN/AK26/1982/G2P[4]	-	-	**86.6**	94.3	92.1	-	92.4	**83.8**	95.0	-	93.9
RVA/Human-tc/JPN/KUN/1980/G2P[4]	-	-	**89.3**	93.6*	93.8	**85.1***	91.6*	**82.9***	91.8*	90.0	91.1*
RVA/Human-wt/JPN/86Y1329/1986/G2P[4]	-	-	-	94.1*	-	-	**85.5***	**79.3***	**87.4***	-	-
RVA/Human-wt/BGD/MMC6/2005/G2P[4]	-	-	-	97.6	95.7	-	97.5	-	97.5	**89.3**	-
RVA/Human-wt/BGN/M334/2013/G2P[4]	-	-	-	-	-	-	97.7	-	-	-	-
RVA/Human-wt/USA/LB2764/2005/G2P[4]	-	-	97.4	96.2*	94.5*	93.5*	94.7*	93.6*	92.9	**84.6***	92.2*
RVA/Human-wt/USA/LB2772/2005/G2P[4]	-	-	-	-	-	91.8*	-	-	-	-	-
RVA/Human-wt/AUS/CK20047/2011/G2P[4]	-	-	-	-	-	94.1*	-	-	-	-	-
RVA/Human-wt/AUS/CK20001/1977/G2P[4]	-	-	**89.6**	-	91.4*	-	**89.5**	**81.3**	-	**84.8***	-
RVA/Human-wt/AUS/CK20033/2006/G2P[4]	-	-	-	-	**86.4**	-	-	-	-	-	-
RVA/Human-wt/JPN/B110119/2011/G2P[4]	-	-	-	-	94.4	-	**88.9**	**87.8**	-	**78.0**	-
RVA/Human-wt/JPN/B110056/2011/G2P[4]	-	-	-	-	**85.6**	-	92.2	**89.0**	-	**87.6**	-
RVA/Human-wt/IND/116E3D/1993/G2P[4]	-	-	-	-	-	-	-	-	-	**79.3**	-
RVA/Human-wt/AUS/V233/1999/G2P[4]	-	-	-	-	-	-	-	-	-	**78.2**	-
RVA/Human-wt/USA/2007769964/2007/G2P[4]	-	-	-	-	-	-	**89.6**	-	-	-	-
RVA/Human-wt/ITA/PAI11/1996/G2P[4]	-	-	-	-	-	-	92.0	-	-	90.1	-
RVA/Human-wt/BGD/MMC88/2005/G2P[4]	-	-	97.3	-	**87.3**	98.5	-	-	-	**88.5**	97.4
RVA/Human-wt/BGD/J303/2010/G2P[4]	-	-	-	97.5	**87.1**	98.3	-	-	-	**88.2**	97.5
RVA/Human-wt/CAN/RT128-07/2008/G2P[4]	-	-	94.0	96.1*	94.2*	**87.1***	-	-	-	**69.2***	**73.0***
RVA/Human-wt/BGN/J263/2010/G2P[4]	-	-	93.6	97.5	**87.1**	98.5	97.8	98.5	97.2	96.6	-
RVA/Human-wt/BRA/RJ12225/2006/G2P[4]	-	-	97.3	98.0	96.0*	92.6*	-	-	-	-	-
RVA/Human-wt/BRA/MA14286/2007/G2P[4]	-	-	97.3	98.0	95.8*	91.0*	-	-	-	-	-
RVA/Human-wt/BRA/SC19868/2011/G2P[4]	-	-	-	97.8	94.5*	95.5	-	-	-	-	-
RVA/Human-wt/GHA/Ghan-148/2007/G2P[4]	-	-	-	-	94.2*	**85.5***	-	-	-	-	-
RVA/Human-wt/BGN/J253/2010/G2P[4]	-	-	-	-	93.7	-	-	-	-	-	-
RVA/Human-wt/BRA/ES16238/2009/G2P[4]	-	-	-	96.9	**85.8***	94.3*	-	-	-	-	-
RVA/Human-wt/COD/DRC86/2003/G8P[6]	-	-	96.8	97.6	96.6	96.0	96.4	96.8	97.6	90.8	97.6
RVA/Human-wt/IND/RV09/2009/G9P[4]	-	-	-	-	-	-	98.4	-	-	-	-
RVA/Human-wt/BRA/QUI-154-F1/2010/G12P[6]	-	-	-	-	-	-	94.3*	90.3*	**88.8***	**87.2**	96.5
RVA/Human-wt/BGD/RV161/2000/G12P[6]	-	-	98.1	98.0	96.3	97.4	96.9	97.7	97.6	-	98.5
RVA/Human-wt/CHN/TB-Chen/1996/G2P[4]	-	-	**89.8**	96.2	93.4	**87.0**	96.1	**86.2**	97.1	**89.4**	97.5
RVA/Human-wt/MWI/MW670/1999/G4P[8]	-	**87.5**	-	-	-	-	-	-	-	-	-
RVA/Human-wt/MWI/OP530/1999/G4P[8]	-	**87.4**	-	-	-	-	-	-	-	-	-
RVA/Human-wt/BEL/B1711/2002/G6P[6]	-	-	-	-	-	97.1	96.1	97.0	97.5	**89.0**	98.0
RVA/Human-wt/IND/RV11/2011/G9P[4]	-	-	-	-	-	-	98.5	-	-	-	-
RVA/Human-tc/USA/WI61/1983/G9P[8]	-	92.0	-	-	-	-	-	-	-	-	-
RVA/Human-wt/MWI/F45/1987/G9P[8]	-	91.4	-	-	-	-	-	-	-	-	-
RVA/Human-wt/PRY/469/2000/G9P[8]	-	98.0	-	-	-	-	-	-	-	-	-
RVA/Human-wt/THA/CU616-TK/09/2009/G9P[8]	-	96.7	-	-	-	-	-	-	-	-	-
RVA/Human-wt/PHL/L26/1987/G12P[4]	-	-	**86.5***	96.9	-	**76.6***	-	-	95.6	-	-
RVA/Human-tc/KEN/KDH651/2010/G12P[8]	-	95.2	-	-	-	-	-	-	-	-	-
RVA/Human-wt/BRA/RJ12419/2006/G12P[8]	-	97.4	-	-	-	-	-	-	-	-	-

*Nucleotide identity is based on partial ORF sequences and/or incomplete sequences.

(B)

	Nucleotide identity (%)										
**IAL-R3123**	**VP7**	**VP4**	**VP1**	**VP2**	**VP3**	**VP6**	**NSP1**	**NSP2**	**NSP3**	**NSP4**	**NSP5**
RVA/Human-wt/BRA/IAL-R3122/2013/G1P[8]	100	99.9	99.8	99.9	99.9	99.8	100	99.9	99.9	99.8	99.8
RVA/Human-wt/BRA/IAL-R3165/2013/G1P[8]	100	99.8	99.3	99.8	99.9	99.8	99.8	99.9	99.7	100	100
RVA/Human-wt/BRA/IAL-R3172/2013/G1P[8]	100	99.8	99.5	99.8	99.9	99.8	99.8	99.8	99.6	99.8	99.8
RVA/Human-wt/MWI/BID2BS/2013/G1P[8]	**85.1**	94.4	94.0	97.2	95.8	98.0	97.1	97.2	97.3	91.4	97.6
RVA/Human-wt/JPN/OH3625/2012/G1P[8]	-	-	-	-	-	-	-	-	-	**86.4**	-
RVA/Human-wt/JPN/KN041/2012/G1P[8]	-	-	-	-	-	-	-	-	-	**84.9**	-
RVA/Human-wt/JPN/HC12016/2012/G1P[8]	93.5*	99.2	99.0	98.3*	97.8*	94.0*	-	91.8*	92.7*	90.9*	**89.2***
RVA/Human-wt/THA/PCB-180/2013/G1P[8]	98.3	97.7	99.3	99.1	98.9	99.0	-	98.2*	99.1	97.3	97.1*
RVA/Human-wt/PHI/TGO12-004/2012/G1P[8]	98.6	-	-	-	-	97.9	-	97.3	99.5	98.5	-
RVA/Human-wt/PHI/TGO12-045/2012/G1P[8]	-	98.7	-	99.6	98.8	-	99.6	-	-	-	99.2
RVA/Human-wt/THA/SKT-109/2013/G1P[8]	-	-	-	-	-	-	99.6	-	-	-	-
RVA/Human-wt/JPN/To14-41/2014/G1P[8]	-	-	-	-	-	-	97.8*	-	-	-	-
RVA/Human-wt/VNM/SP026/2012/G1P[8]	90.5*	97.7	98.0	97.4*	96.6*	96.1*	96.4*	94.1*	94.9	92.4*	94.8*
RVA/Human-wt/BGD/Dhaka16/2003/G1P[8]	96.3	97.4	-	-	-	-	-	-	-	-	-
RVA/Vaccine/USA/Rotarix-AROLA490AB/1988/G1P[8]	**89.7***	**89.4**	-	-	-	-	-	-	-	-	-
RVA/Vaccine/USA/RotaTeq-WI79-9/1992/G1P7[5]	91.6	91.6	-	-	-	-	-	-	-	-	-

RVA/Human-wt/BEL/B74-02/2002/G1P[8]	**89.1***	-	-	-	-	-	-	-	-	-	-
RVA/Human-wt/USA/2007719635/2007/G1P[8]	94.7*	-	-	-	-	-	-	-	-	-	-
RVA/Human-wt/USA/CU957-KK/11/2011/G1P[8]	**87.1***	-	-	-	-	-	-	-	-	-	-
RVA/Human-tc/USA/D/1974/G1P[8]	**84.5***	-	-	-	-	-	-	-	-	-	-
RVA/Human-wt/USA/Wa/1974/G1P[8]	-	**89.6**	-	-	-	-	-	-	-	-	-
RVA/Human-wt/BEL/BE00094/2009/G1P[8]	-	95.4	-	-	-	-	-	-	-	-	-
RVA/Human-tc/USA/K54/xxxx/G1P[x]	**84.9***	-	-	-	-	-	-	-	-	-	-
RVA/Human-wt/JPN/AU19/xxxx/G1P[x]	**78.8***	-	-	-	-	-	-	-	-	-	-
RVA/Bovine-wt/XXX/T449/xxxx/G1P[x]	**85.7**	-	-	-	-	-	-	-	-	-	-
RVA/Human-wt/BRA/QUI-150-F1/2010/G1P[8]	**88.2***	-	-	-	-	-	-	-	-	-	-
RVA/Human-wt/BRA/R321-2004/2004/G1P[x]	91.0	-	-	-	-	-	-	-	-	-	-
RVA/Human-tc/BRA/R70/1997/G1P[9]	91.0*	-	-	-	-	-	-	-	-	-	-
RVA/Human-wt/PAR/Py03IPN144/2002-2005/G1P[8]	**85.7***	-	-	-	-	-	-	-	-	-	-
RVA/Pig-tc/VEN/C60/xxxx/G1P[x]	**84.5**	-	-	-	-	-	-	-	-	-	-
RVA/Human-wt/ISR/Isr-56/1995/G1P[x]	**85.1***	-	-	-	-	-	-	-	-	-	-
RVA/Human-wt/BRA/Brz-6/1995/G1P[x]	**84.1***	-	-	-	-	-	-	-	-	-	-
RVA/Human-wt/JPN/87Y1397/xxxx/G1P[8]	92.9	-	-	-	-	-	-	-	-	-	-
RVA/Human-wt/KOR/Kor-64/1988/G1P[x]	**85.4***	-	-	-	-	-	-	-	-	-	-
RVA/Human-wt/ITA/PA10/90/1990/G1P[8]	**83.5***	-	-	-	-	-	-	-	-	-	-
RVA/Human-wt/ITA/PA78/89/1989/G1P[8]	**84.0***	-	-	-	-	-	-	-	-	-	-
RVA/Human-wt/URY/Mvd9606/1996/G1P[8]	94.1	-	-	-	-	-	-	-	-	-	-
RVA/Human-wt/ITA/PA33/99/1999/G1P[8]	**83.3***	-	-	-	-	-	-	-	-	-	-
RVA/Human-wt/NCA/9J/2010/G1P[8]	**88.4***	97.2	-	-	-	-	-	-	-	-	-
RVA/Human-wt/AUS/CK00083/2008/G1P[8]	92.5*	-	-	-	-	-	-	-	-	-	-
RVA/Human-wt/URY/Mvd9806/1996/G1P[8]	97.1	-	-	-	-	-	-	-	-	-	-
RVA/Human-wt/ITA/PA258/97/1997/G1P[8]	**82.7***	-	-	-	-	-	-	-	-	-	-
RVA/Human-wt/AUS/WAPC1740/2013/G3P[8]	-	-	-	-	-	-	99.6	99.8	-	-	-
RVA/Human-wt/AUS/WAPC2016/2014/G3P[8]	-	-	-	99.3	98.7	99.7	-	-	-	-	99.3
RVA/Human-wt/ESP/SS98244047/2015/G3P[8]	-	-	-	-	-	-	-	99.5	99.4	97.8	99.0
RVA/Human-wt/JPN/IS1078/2015/G3P[8]	-	-	-	-	-	99.2	99.3	-	99.6	97.6	-
RVA/Human-wt/USA/3000390639/2015/G3P[8]	-	-	98.6	-	-	-	-	-	-	-	-
RVA/Human-wt/HUN/ERN8263/2015/G3P[8]	-	-	98.8	-	-	-	-	-	-	-	-
RVA/Human-wt/THA/SKT-289/2013/G3P[8]	-	98.4	-	99.2	99.2	-	-	-	-	-	-
RVA/Human-wt/USA/DC23/1976/G3P[8]	-	**89.6***	-	-	-	-	-	-	-	-	-
RVA/Human-wt/AUS/D388/2013/G3P[8]	-	98.8	-	-	-	-	-	-	-	-	-
RVA/Human-wt/JPN/ITO/1981/G3P[8]	-	**88.8**	-	-	-	-	-	-	-	-	-
RVA/Human-wt/BEL/BE87/2011/G2P[4]	-	-	-	-	-	-	91.6*	**87.5***	-	**49.9***	**72.6***
RVA/Human-wt/ITA/PA108/2007/G2P[4]	-	-	-	-	95.9	98.6	97.8*	-	-	-	-
RVA/Human-wt/JPN/07Y003/2007/G2P[4]	-	-	-	-	-	-	90.6*	-	-	-	-
RVA/Human-wt/BGN/J306/2010/G2P[4]	-	-	-	-	-	-	98.6	-	-	-	-
RVA/Human-wt/BRA/QUI-60-F1/2008/G2P[4]	-	-	-	-	-	-	96.1*	94.8*	91.2*	94.8	96.2
RVA/Human-wt/BRA/SE19300/2010/G2P[4]	-	-	-	-	-	-	96.1*	90.8*	91.0*	**79.7***	90.3*
RVA/Human-wt/BRA/MS11142/2005/G2P[4]	-	-	97.3	-	-	-	96.6*	91.4*	91.9*	**78.5***	91.7*
RVA/Human-wt/JPN/90A009/1990/G2P[4]	-	-	-	-	-	-	-	-	**89.4***	**82.8***	-
RVA/Human-wt/JPN/90H094/1990/G2P[4]	-	-	-	92.9*	-	-	-	-	-	-	-
RVA/Human-wt/JPN/83A001/1983/G2P[4]	-	-	**88.4***	**89.5***	91.0*	**81.1***	**84.4***	**76.6**	**86.3***	**77.7***	**81.9***
RVA/Human-wt/JPN/89Y1520/1989/G2P[4]	-	-	**87.1***	-	91.1*	**80.7***	**85.5***	-	**87.5***	**84.4***	**85.7***
RVA/Human-tc/KEN/D205/1989/G2P[4]	-	-	**86.4**	93.6	-	-	95.0	96.1	96.5	90.1]2	93.5
RVA/Human-wt/KEN/AK26/1982/G2P[4]	-	-	**86.7**	94.3	92.0	-	92.4	**83.8**	95.1	-	93.8
RVA/Human-tc/JPN/KUN/1980/G2P[4]	-	-	**89.4**	93.6*	93.8	**85.1***	91.6*	**82.9***	91.8*	90.1	91.2
RVA/Human-wt/JPN/86Y1329/1986/G2P[4]	-	-	-	94.2*	-	-	**85.5***	**79.3***	**87.4***	-	-
RVA/Human-wt/BGD/MMC6/2005/G2P[4]	-	-	-	97.6	95.7	-	97.5	-	97.6	**89.4**	-
RVA/Human-wt/BGN/M334/2013/G2P[4]	-	-	-	-	-	-	97.7	-	-	-	-
RVA/Human-wt/USA/LB2764/2005/G2P[4]	-	-	97.4	96.2*	94.4*	93.5*	94.7*	93.6*	92.9	**84.8***	92.4*
RVA/Human-wt/USA/LB2772/2005/G2P[4]	-	-	-	-	-	91.8*	-	-	-	-	-
RVA/Human-wt/AUS/CK20047/2011/G2P[4]	-	-	-	-	-	94.1*	-	-	-	-	-
RVA/Human-wt/AUS/CK20001/1977/G2P[4]	-	-	**89.7**	-	91.3*	-	**89.5**	**81.3**	-	**84.9***	-
RVA/Human-wt/AUS/CK20033/2006/G2P[4]	-	-	-	-	**86.4**	-	-	-	-	-	-
RVA/Human-wt/JPN/B110119/2011/G2P[4]	-	-	-	-	94.4	-	**88.9**	**87.8**	-	**78.0**	-
RVA/Human-wt/JPN/B110056/2011/G2P[4]	-	-	-	-	**85.6**	-	92.2	**89.1**	-	**87.7**	-
RVA/Human-wt/IND/116E3D/1993/G2P[4]	-	-	-	-	-	-	-	-	-	**79.4**	-
RVA/Human-wt/AUS/V233/1999/G2P[4]	-	-	-	-	-	-	-	-	-	**78.4**	-
RVA/Human-wt/USA/2007769964/2007/G2P[4]	-	-	-	-	-	-	**89.6**	-	-	-	-
RVA/Human-wt/ITA/PAI11/1996/G2P[4]	-	-	-	-	-	-	92.0	-	-	90.2	-
RVA/Human-wt/BGD/MMC88/2005/G2P[4]	-	-	97.2	-	**87.3**	98.5	-	-	-	**88.6**	97.3
RVA/Human-wt/BGD/J303/2010/G2P[4]	-	-	-	97.5	**87.1**	98.3	-	-	-	**88.4**	97.4
RVA/Human-wt/CAN/RT128-07/2008/G2P[4]	-	-	94.1	96.1*	94.2*	**87.1***	-	-	-	**69.2***	**73.1***
RVA/Human-wt/BGN/J263/2010/G2P[4]	-	-	93.6	97.5	**87.1**	98.5	97.8	98.6	97.3	96.8	-
RVA/Human-wt/BRA/RJ12225/2006/G2P[4]	-	-	97.3	98.0	95.9*	92.6*	-	-	-	-	-
RVA/Human-wt/BRA/MA14286/2007/G2P[4]	-	-	97.3	98.0	95.8*	91.0*	-	-	-	-	-
RVA/Human-wt/BRA/SC19868/2011/G2P[4]	-	-	-	97.8	94.5*	95.5	-	-	-	-	-

RVA/Human-wt/GHA/Ghan-148/2007/G2P[4]	-	-	-	-	94.1*	**85.5***	-	-	-	-	-
RVA/Human-wt/BGN/J253/2010/G2P[4]	-	-	-	-	93.7	-	-	-	-	-	-
RVA/Human-wt/BRA/ES16238/2009/G2P[4]	-	-	-	96.9	**85.8***	94.3*	-	-	-	-	-
RVA/Human-wt/COD/DRC86/2003/G8P[6]	-	-	96.8	97.6	96.6	96.0	96.4	96.8	97.7	90.9	97.5
RVA/Human-wt/IND/RV09/2009/G9P[4]	-	-	-	-	-	-	98.4	-	-	-	-
RVA/Human-wt/BRA/QUI-154-F1/2010/G12P[6]	-	-	-	-	-	-	94.3*	90.4*	**88.8***	**87.3**	96.4
RVA/Human-wt/BGD/RV161/2000/G12P[6]	-	-	98.0	98.0	96.3	97.4	96.9	97.7	97.7	-	98.4
RVA/Human-wt/CHN/TB-Chen/1996/G2P[4]	-	-	**89.9**	96.2	93.3	**87.1**	96.1	**86.2**	97.2	**89.6**	97.4
RVA/Human-wt/MWI/MW670/1999/G4P[8]	-	**87.6**	-	-	-	-	-	-	-	-	-
RVA/Human-wt/MWI/OP530/1999/G4P[8]	-	**87.5**	-	-	-	-	-	-	-	-	-
RVA/Human-wt/BEL/B1711/2002/G6P[6]	-	-	-	-	-	97.1	96.1	97.0	97.6	**89.2**	97.9
RVA/Human-wt/IND/RV11/2011/G9P[4]	-	-	-	-	-	-	98.5	-	-	-	-
RVA/Human-tc/USA/WI61/1983/G9P[8]	-	92.1	-	-	-	-	-	-	-	-	-
RVA/Human-wt/MWI/F45/1987/G9P[8]	-	91.5	-	-	-	-	-	-	-	-	-
RVA/Human-wt/PRY/469/2000/G9P[8]	-	98.0	-	-	-	-	-	-	-	-	-
RVA/Human-wt/THA/CU616-TK/09/2009/G9P[8]	-	96.7	-	-	-	-	-	-	-	-	-
RVA/Human-wt/PHL/L26/1987/G12P[4]	-	-	**86.6***	96.9	-	**76.7***	-	-	95.7	-	-
RVA/Human-tc/KEN/KDH651/2010/G12P[8]	-	95.3	-	-	-	-	-	-	-	-	-
RVA/Human-wt/BRA/RJ12419/2006/G12P[8]	-	97.4	-	-	-	-	-	-	-	-	-

*Nucleotide identity is based on partial ORF sequences and/or incomplete sequences.

(C)

	Nucleotide identity (%)										
**IAL-R3165**	**VP7**	**VP4**	**VP1**	**VP2**	**VP3**	**VP6**	**NSP1**	**NSP2**	**NSP3**	**NSP4**	**NSP5**
RVA/Human-wt/BRA/IAL-R3122/2013/G1P[8]	100	99.8	99.8	99.8	99.9	100	99.8	99.9	99.6	99.8	99.8
RVA/Human-wt/BRA/IAL-R3123/2013/G1P[8]	100	99.8	99.3	99.8	99.9	99.8	99.8	99.8	99.7	100	100
RVA/Human-wt/BRA/IAL-R3172/2013/G1P[8]	100	99.9	99.4	99.8	99.9	99.8	99.9	99.9	99.9	99.8	99.8
RVA/Human-wt/MWI/BID2BS/2013/G1P[8]	**85.1**	94.5	93.7	97.3	95.8	98.0	97.3	97.2	97.6	91.4	97.6
RVA/Human-wt/JPN/OH3625/2012/G1P[8]	-	-	-	-	-	-	-	-	-	**86.4**	-
RVA/Human-wt/JPN/KN041/2012/G1P[8]	-	-	-	-	-	-	-	-	-	**84.9**	-
RVA/Human-wt/JPN/HC12016/2012/G1P[8]	93.5*	99.3	98.8	98.4*	97.9*	93.8*	-	91.8*	93.0*	90.9*	**89.2***
RVA/Human-wt/THA/PCB-180/2013/G1P[8]	98.3	97.8	99.1	99.1	98.9	98.8	-	98.3*	99.4	97.3	97.1*
RVA/Human-wt/PHI/TGO12-004/2012/G1P[8]	98.6	-	-	-	-	97.9	-	97.4	99.8	98.5	-
RVA/Human-wt/PHI/TGO12-045/2012/G1P[8]	-	98.8	-	99.7	98.9	-	99.7	-	-	-	99.2
RVA/Human-wt/THA/SKT-109/2013/G1P[8]	-	-	-	-	-	-	99.8	-	-	-	-
RVA/Human-wt/JPN/To14-41/2014/G1P[8]	-	-	-	-	-	-	98.0*	-	-	-	-
RVA/Human-wt/VNM/SP026/2012/G1P[8]	90.5*	97.8	97.9	97.4*	96.7*	96.1*	96.6*	94.2*	95.2	92.4*	94.8*
RVA/Human-wt/BGD/Dhaka16/2003/G1P[8]	96.3	97.5	-	-	-	-	-	-	-	-	-
RVA/Vaccine/USA/Rotarix-AROLA490AB/1988/G1P[8]	**89.7***	**89.4**	-	-	-	-	-	-	-	-	-
RVA/Vaccine/USA/RotaTeq-WI79-9/1992/G1P7[5]	91.6	91.7	-	-	-	-	-	-	-	-	-
RVA/Human-wt/BEL/B74-02/2002/G1P[8]	**89.1***	-	-	-	-	-	-	-	-	-	-
RVA/Human-wt/USA/2007719635/2007/G1P[8]	94.7*	-	-	-	-	-	-	-	-	-	-
RVA/Human-wt/USA/CU957-KK/11/2011/G1P[8]	**87.1***	-	-	-	-	-	-	-	-	-	-
RVA/Human-tc/USA/D/1974/G1P[8]	**84.5***	-	-	-	-	-	-	-	-	-	-
RVA/Human-wt/USA/Wa/1974/G1P[8]	-	**89.7**	-	-	-	-	-	-	-	-	-
RVA/Human-wt/BEL/BE00094/2009/G1P[8]	-	95.5	-	-	-	-	-	-	-	-	-
RVA/Human-tc/USA/K54/xxxx/G1P[x]	**84.9***	-	-	-	-	-	-	-	-	-	-
RVA/Human-wt/JPN/AU19/xxxx/G1P[x]	**78.8***	-	-	-	-	-	-	-	-	-	-
RVA/Bovine-wt/XXX/T449/xxxx/G1P[x]	**85.7**	-	-	-	-	-	-	-	-	-	-
RVA/Human-wt/BRA/QUI-150-F1/2010/G1P[8]	**88.2***	-	-	-	-	-	-	-	-	-	-
RVA/Human-wt/BRA/R321-2004/2004/G1P[x]	91.0	-	-	-	-	-	-	-	-	-	-
RVA/Human-tc/BRA/R70/1997/G1P[9]	91.0*	-	-	-	-	-	-	-	-	-	-
RVA/Human-wt/PAR/Py03IPN144/2002-2005/G1P[8]	**85.7***	-	-	-	-	-	-	-	-	-	-
RVA/Pig-tc/VEN/C60/xxxx/G1P[x]	**84.5**	-	-	-	-	-	-	-	-	-	-
RVA/Human-wt/ISR/Isr-56/1995/G1P[x]	**85.1***	-	-	-	-	-	-	-	-	-	-
RVA/Human-wt/BRA/Brz-6/1995/G1P[x]	**84.1***	-	-	-	-	-	-	-	-	-	-
RVA/Human-wt/JPN/87Y1397/xxxx/G1P[8]	92.9	-	-	-	-	-	-	-	-	-	-
RVA/Human-wt/KOR/Kor-64/1988/G1P[x]	**85.4***	-	-	-	-	-	-	-	-	-	-
RVA/Human-wt/ITA/PA10/90/1990/G1P[8]	**83.5***	-	-	-	-	-	-	-	-	-	-
RVA/Human-wt/ITA/PA78/89/1989/G1P[8]	**84.0***	-	-	-	-	-	-	-	-	-	-
RVA/Human-wt/URY/Mvd9606/1996/G1P[8]	94.1	-	-	-	-	-	-	-	-	-	-
RVA/Human-wt/ITA/PA33/99/1999/G1P[8]	**83.3***	-	-	-	-	-	-	-	-	-	-
RVA/Human-wt/NCA/9J/2010/G1P[8]	**88.4***	97.2	-	-	-	-	-	-	-	-	-
RVA/Human-wt/AUS/CK00083/2008/G1P[8]	92.5*	-	-	-	-	-	-	-	-	-	-
RVA/Human-wt/URY/Mvd9806/1996/G1P[8]	97.1	-	-	-	-	-	-	-	-	-	-
RVA/Human-wt/ITA/PA258/97/1997/G1P[8]	**82.7***	-	-	-	-	-	-	-	-	-	-
RVA/Human-wt/AUS/WAPC1740/2013/G3P[8]	-	-	-	-	-	-	99.7	99.6	-	-	-
RVA/Human-wt/AUS/WAPC2016/2014/G3P[8]	-	-	-	99.4	98.8	99.5	-	-	-	-	99.3
RVA/Human-wt/ESP/SS98244047/2015/G3P[8]	-	-	-	-	-	-	-	99.5	99.7	97.8	99.0
RVA/Human-wt/JPN/IS1078/2015/G3P[8]	-	-	-	-	-	99.1	99.4	-	99.9	97.6	-

	VP7	VP4	VP1	VP2	VP3	VP6	NSP1	NSP2	NSP3	NSP4	NSP5
RVA/Human-wt/USA/3000390639/2015/G3P[8]	-	-	98.4	-	-	-	-	-	-	-	-
RVA/Human-wt/HUN/ERN8263/2015/G3P[8]	-	-	98.6	-	-	-	-	-	-	-	-
RVA/Human-wt/THA/SKT-289/2013/G3P[8]	-	98.5	-	99.2	99.3	-	-	-	-	-	-
RVA/Human-wt/USA/DC23/1976/G3P[8]	-	**89.7***	-	-	-	-	-	-	-	-	-
RVA/Human-wt/AUS/D388/2013/G3P[8]	-	98.9	-	-	-	-	-	-	-	-	-
RVA/Human-wt/JPN/ITO/1981/G3P[8]	-	**88.8**	-	-	-	-	-	-	-	-	-
RVA/Human-wt/BEL/BE87/2011/G2P[4]	-	-	-	-	-	-	91.7*	**87.5***	-	**49.9***	**72.6***
RVA/Human-wt/ITA/PA108/2007/G2P[4]	-	-	-	-	96.0	98.6	98.0*	-	-	-	-
RVA/Human-wt/JPN/07Y003/2007/G2P[4]	-	-	-	-	-	-	90.8*	-	-	-	-
RVA/Human-wt/BGN/J306/2010/G2P[4]	-	-	-	-	-	-	98.7	-	-	-	-
RVA/Human-wt/BRA/QUI-60-F1/2008/G2P[4]	-	-	-	-	-	-	96.2*	94.9*	91.5*	94.8	96.2
RVA/Human-wt/BRA/SE19300/2010/G2P[4]	-	-	-	-	-	-	96.2*	90.9*	91.1*	**79.7***	90.3*
RVA/Human-wt/BRA/MS11142/2005/G2P[4]	-	-	97.0	-	-	-	96.7*	91.5*	92.2*	**78.5***	91.7*
RVA/Human-wt/JPN/90A009/1990/G2P[4]	-	-	-	-	-	-	-	-	**89.7***	**82.8***	-
RVA/Human-wt/JPN/90H094/1990/G2P[4]	-	-	-	92.9*	-	-	-	-	-	-	-
RVA/Human-wt/JPN/83A001/1983/G2P[4]	-	-	**88.1***	**89.6***	91.1*	**81.0***	**84.5***	76.7	**86.4***	**77.7***	**81.9***
RVA/Human-wt/JPN/89Y1520/1989/G2P[4]	-	-	**86.7***	-	91.2*	**80.6***	**85.5***	-	**87.8***	**84.4***	**85.7***
RVA/Human-tc/KEN/D205/1989/G2P[4]	-	-	**86.1**	93.7	-	-	95.2	96.0	96.8	90.1]2	93.5
RVA/Human-wt/KEN/AK26/1982/G2P[4]	-	-	**86.2**	94.4	92.1	-	92.5	**83.9**	95.4	-	93.8
RVA/Human-tc/JPN/KUN/1980/G2P[4]	-	-	**89.0**	93.6*	93.9	**85.1***	91.7*	**83.0***	92.1*	90.1	91.2
RVA/Human-wt/JPN/86Y1329/1986/G2P[4]	-	-	-	94.1*	-	-	**85.7***	**79.3***	**87.7***	-	-
RVA/Human-wt/BGD/MMC6/2005/G2P[4]	-	-	-	97.7	95.7	-	97.6	-	97.9	**89.4**	-
RVA/Human-wt/BGN/M334/2013/G2P[4]	-	-	-	-	-	-	97.8	-	-	-	-
RVA/Human-wt/USA/LB2764/2005/G2P[4]	-	-	97.2	96.3*	94.5*	93.5*	94.8*	93.7*	93.0	**84.8***	92.4*
RVA/Human-wt/USA/LB2772/2005/G2P[4]	-	-	-	-	-	91.8*	-	-	-	-	-
RVA/Human-wt/AUS/CK20047/2011/G2P[4]	-	-	-	-	-	94.1*	-	-	-	-	-
RVA/Human-wt/AUS/CK20001/1977/G2P[4]	-	-	**89.4**	-	91.4*	-	**89.7**	**81.4**	-	**84.9***	-
RVA/Human-wt/AUS/CK20033/2006/G2P[4]	-	-	-	-	**86.4**	-	-	-	-	-	-
RVA/Human-wt/JPN/B110119/2011/G2P[4]	-	-	-	-	94.4	-	**89.0**	**87.9**	-	**78.0**	-
RVA/Human-wt/JPN/B110056/2011/G2P[4]	-	-	-	-	**85.6**	-	92.4	**89.1**	-	**87.7**	-
RVA/Human-wt/IND/116E3D/1993/G2P[4]	-	-	-	-	-	-	-	-	-	**79.4**	-
RVA/Human-wt/AUS/V233/1999/G2P[4]	-	-	-	-	-	-	-	-	-	**78.4**	-
RVA/Human-wt/USA/2007769964/2007/G2P[4]	-	-	-	-	-	-	**89.7**	-	-	-	-
RVA/Human-wt/ITA/PAI11/1996/G2P[4]	-	-	-	-	-	-	92.1	-	-	90.2	-
RVA/Human-wt/BGD/MMC88/2005/G2P[4]	-	-	97.1	-	**87.3**	98.5	-	-	-	**88.6**	97.3
RVA/Human-wt/BGD/J303/2010/G2P[4]	-	-	-	97.5	**87.1**	98.3	-	-	-	**88.4**	97.4
RVA/Human-wt/CAN/RT128-07/2008/G2P[4]	-	-	94.1	96.1*	94.3*	**87.1***	-	-	-	**69.2***	**73.1***
RVA/Human-wt/BGN/J263/2010/G2P[4]	-	-	93.3	97.6	**87.1**	98.5	97.9	98.6	97.6	96.8	-
RVA/Human-wt/BRA/RJ12225/2006/G2P[4]	-	-	97.0	98.0	96.0*	92.6*	-	-	-	-	-
RVA/Human-wt/BRA/MA14286/2007/G2P[4]	-	-	97.0	98.1	95.9*	91.0*	-	-	-	-	-
RVA/Human-wt/BRA/SC19868/2011/G2P[4]	-	-	-	97.9	94.5*	95.5	-	-	-	-	-
RVA/Human-wt/GHA/Ghan-148/2007/G2P[4]	-	-	-	-	94.2*	**85.5***	-	-	-	-	-
RVA/Human-wt/BGN/J253/2010/G2P[4]	-	-	-	-	93.7	-	-	-	-	-	-
RVA/Human-wt/BRA/ES16238/2009/G2P[4]	-	-	-	97.0	**85.8***	94.3*	-	-	-	-	-
RVA/Human-wt/COD/DRC86/2003/G8P[6]	-	-	96.5	97.7	96.6	96.0	96.5	96.9	98.0	90.9	97.5
RVA/Human-wt/IND/RV09/2009/G9P[4]	-	-	-	-	-	-	98.5	-	-	-	-
RVA/Human-wt/BRA/QUI-154-F1/2010/G12P[6]	-	-	-	-	-	-	94.5*	90.4*	**88.9***	**87.3**	96.4
RVA/Human-wt/BGD/RV161/2000/G12P[6]	-	-	97.8	98.0	96.4	97.4	97.0	97.8	98.0	-	98.4
RVA/Human-wt/CHN/TB-Chen/1996/G2P[4]	-	-	**89.4**	96.3	93.4	**87.0**	96.2	**86.3**	97.5	**89.6**	97.4
RVA/Human-wt/MWI/MW670/1999/G4P[8]	-	**87.6**	-	-	-	-	-	-	-	-	-
RVA/Human-wt/MWI/OP530/1999/G4P[8]	-	**87.6**	-	-	-	-	-	-	-	-	-
RVA/Human-wt/BEL/B1711/2002/G6P[6]	-	-	-	-	-	97.1	96.2	97.1	97.9	**89.2**	97.9
RVA/Human-wt/IND/RV11/2011/G9P[4]	-	-	-	-	-	-	98.7	-	-	-	-
RVA/Human-tc/USA/WI61/1983/G9P[8]	-	92.2	-	-	-	-	-	-	-	-	-
RVA/Human-wt/MWI/F45/1987/G9P[8]	-	91.5	-	-	-	-	-	-	-	-	-
RVA/Human-wt/PRY/469/2000/G9P[8]	-	98.1	-	-	-	-	-	-	-	-	-
RVA/Human-wt/THA/CU616-TK/09/2009/G9P[8]	-	96.8	-	-	-	-	-	-	-	-	-
RVA/Human-wt/PHL/L26/1987/G12P[4]	-	-	**86.3***	96.9	-	**76.6***	-	-	96.0	-	-
RVA/Human-tc/KEN/KDH651/2010/G12P[8]	-	95.4	-	-	-	-	-	-	-	-	-
RVA/Human-wt/BRA/RJ12419/2006/G12P[8]	-	97.5	-	-	-	-	-	-	-	-	-

*Nucleotide identity is based on partial ORF sequences and/or incomplete sequences.

(D)

	Nucleotide identity (%)										
**IAL-R3172**	**VP7**	**VP4**	**VP1**	**VP2**	**VP3**	**VP6**	**NSP1**	**NSP2**	**NSP3**	**NSP4**	**NSP5**
RVA/Human-wt/BRA/IAL-R3122/2013/G1P[8]	100	99.8	99.6	99.7	99.9	99.8	99.8	99.8	99.7	100	99.7
RVA/Human-wt/BRA/IAL-R3123/2013/G1P[8]	100	99.8	99.5	99.8	99.9	100	99.8	99.9	99.6	99.8	99.8
RVA/Human-wt/BRA/IAL-R3165/2013/G1P[8]	100	99.9	99.4	99.8	99.9	99.8	99.9	99.9	99.9	99.8	99.8
RVA/Human-wt/MWI/BID2BS/2013/G1P[8]	**85.1**	94.5	94.0	97.2	95.8	98.0	97.3	97.3	97.5	91.3	97.5
RVA/Human-wt/JPN/OH3625/2012/G1P[8]	-	-	-	-	-	-	-	-	-	**86.2**	-

RVA/Human-wt/JPN/KN041/2012/G1P[8]	-	-	-	-	-	-	-	-	-	**84.8**	-
RVA/Human-wt/JPN/HC12016/2012/G1P[8]	93.5*	99.3	99.0	98.4*	97.9*	94.0*	-	91.9*	93.0*	90.8*	**89.3***
RVA/Human-wt/THA/PCB-180/2013/G1P[8]	98.3	97.8	99.3	99.1	98.9	99.0	-	98.4*	99.4	97.2	97.3*
RVA/Human-wt/PHI/TGO12-004/2012/G1P[8]	98.6	-	-	-	-	97.9	-	97.4	99.7	98.4	-
RVA/Human-wt/PHI/TGO12-045/2012/G1P[8]	-	98.8	-	99.6	98.9	-	99.8	-	-	-	99.1
RVA/Human-wt/THA/SKT-109/2013/G1P[8]	-	-	-	-	-	-	99.8	-	-	-	-
RVA/Human-wt/JPN/To14-41/2014/G1P[8]	-	-	-	-	-	-	98.0*	-	-	-	-
RVA/Human-wt/VNM/SP026/2012/G1P[8]	90.5*	97.8	98.1	97.5*	96.6*	96.1*	96.6*	94.2*	95.2	92.2*	94.9*
RVA/Human-wt/BGD/Dhaka16/2003/G1P[8]	96.3	97.4	-	-	-	-	-	-	-	-	-
RVA/Vaccine/USA/Rotarix-AROLA490AB/1988/G1P[8]	**89.7***	**89.4**	-	-	-	-	-	-	-	-	-
RVA/Vaccine/USA/RotaTeq-WI79-9/1992/G1P7[5]	91.6	91.7	-	-	-	-	-	-	-	-	-
RVA/Human-wt/BEL/B74-02/2002/G1P[8]	**89.1***	-	-	-	-	-	-	-	-	-	-
RVA/Human-wt/USA/2007719635/2007/G1P[8]	94.7*	-	-	-	-	-	-	-	-	-	-
RVA/Human-wt/USA/CU957-KK/11/2011/G1P[8]	**87.1***	-	-	-	-	-	-	-	-	-	-
RVA/Human-tc/USA/D/1974/G1P[8]	**84.5***	-	-	-	-	-	-	-	-	-	-
RVA/Human-wt/USA/Wa/1974/G1P[8]	-	**89.7**	-	-	-	-	-	-	-	-	-
RVA/Human-wt/BEL/BE00094/2009/G1P[8]	-	95.5	-	-	-	-	-	-	-	-	-
RVA/Human-tc/USA/K54/xxxx/G1P[x]	**84.9***	-	-	-	-	-	-	-	-	-	-
RVA/Human-wt/JPN/AU19/xxxx/G1P[x]	**78.8***	-	-	-	-	-	-	-	-	-	-
RVA/Bovine-wt/XXX/T449/xxxx/G1P[x]	**85.7**	-	-	-	-	-	-	-	-	-	-
RVA/Human-wt/BRA/QUI-150-F1/2010/G1P[8]	**88.2***	-	-	-	-	-	-	-	-	-	-
RVA/Human-wt/BRA/R321-2004/2004/G1P[x]	91.0	-	-	-	-	-	-	-	-	-	-
RVA/Human-tc/BRA/R70/1997/G1P[9]	91.0*	-	-	-	-	-	-	-	-	-	-
RVA/Human-wt/PAR/Py03IPN144/2002-2005/G1P[8]	**85.7***	-	-	-	-	-	-	-	-	-	-
RVA/Pig-tc/VEN/C60/xxxx/G1P[x]	**84.5**	-	-	-	-	-	-	-	-	-	-
RVA/Human-wt/ISR/Isr-56/1995/G1P[x]	**85.1***	-	-	-	-	-	-	-	-	-	-
RVA/Human-wt/BRA/Brz-6/1995/G1P[x]	**84.1***	-	-	-	-	-	-	-	-	-	-
RVA/Human-wt/JPN/87Y1397/xxxx/G1P[8]	92.9	-	-	-	-	-	-	-	-	-	-
RVA/Human-wt/KOR/Kor-64/1988/G1P[x]	**85.4***	-	-	-	-	-	-	-	-	-	-
RVA/Human-wt/ITA/PA10/90/1990/G1P[8]	**83.5***	-	-	-	-	-	-	-	-	-	-
RVA/Human-wt/ITA/PA78/89/1989/G1P[8]	**84.0***	-	-	-	-	-	-	-	-	-	-
RVA/Human-wt/URY/Mvd9606/1996/G1P[8]	94.1	-	-	-	-	-	-	-	-	-	-
RVA/Human-wt/ITA/PA33/99/1999/G1P[8]	**83.3***	-	-	-	-	-	-	-	-	-	-
RVA/Human-wt/NCA/9J/2010/G1P[8]	**88.4***	97.2	-	-	-	-	-	-	-	-	-
RVA/Human-wt/AUS/CK00083/2008/G1P[8]	92.5*	-	-	-	-	-	-	-	-	-	-
RVA/Human-wt/URY/Mvd9806/1996/G1P[8]	97.1	-	-	-	-	-	-	-	-	-	-
RVA/Human-wt/ITA/PA258/97/1997/G1P[8]	**82.7***	-	-	-	-	-	-	-	-	-	-
RVA/Human-wt/AUS/WAPC1740/2013/G3P[8]	-	-	-	-	-	-	99.8	99.7	-	-	-
RVA/Human-wt/AUS/WAPC2016/2014/G3P[8]	-	-	-	99.3	98.7	99.7	-	-	-	-	99.2
RVA/Human-wt/ESP/SS98244047/2015/G3P[8]	-	-	-	-	-	-	-	99.6	99.6	97.7	98.8
RVA/Human-wt/JPN/IS1078/2015/G3P[8]	-	-	-	-	-	99.2	99.5	-	99.8	97.4	-
RVA/Human-wt/USA/3000390639/2015/G3P[8]	-	-	98.6	-	-	-	-	-	-	-	-
RVA/Human-wt/HUN/ERN8263/2015/G3P[8]	-	-	98.8	-	-	-	-	-	-	-	-
RVA/Human-wt/THA/SKT-289/2013/G3P[8]	-	98.5	-	99.2	99.2	-	-	-	-	-	-
RVA/Human-wt/USA/DC23/1976/G3P[8]	-	**89.7***	-	-	-	-	-	-	-	-	-
RVA/Human-wt/AUS/D388/2013/G3P[8]	-	98.9	-	-	-	-	-	-	-	-	-
RVA/Human-wt/JPN/ITO/1981/G3P[8]	-	**88.8**	-	-	-	-	-	-	-	-	-
RVA/Human-wt/BEL/BE87/2011/G2P[4]	-	-	-	-	-	-	91.8*	**87.6***	-	**49.9***	**72.7***
RVA/Human-wt/ITA/PA108/2007/G2P[4]	-	-	-	-	95.9	98.6	98.0*	-	-	-	-
RVA/Human-wt/JPN/07Y003/2007/G2P[4]	-	-	-	-	-	-	90.8*	-	-	-	-
RVA/Human-wt/BGN/J306/2010/G2P[4]	-	-	-	-	-	-	98.8	-	-	-	-
RVA/Human-wt/BRA/QUI-60-F1/2008/G2P[4]	-	-	-	-	-	-	96.3*	94.8*	91.5*	94.6	96.0
RVA/Human-wt/BRA/SE19300/2010/G2P[4]	-	-	-	-	-	-	96.3*	90.9*	91.1*	**79.6***	90.4*
RVA/Human-wt/BRA/MS11142/2005/G2P[4]	-	-	97.3	-	-	-	96.8*	91.5*	92.2*	**78.4***	91.9*
RVA/Human-wt/JPN/90A009/1990/G2P[4]	-	-	-	-	-	-	-	-	**89.7***	**82.6***	-
RVA/Human-wt/JPN/90H094/1990/G2P[4]	-	-	-	93.0*	-	-	-	-	-	-	-
RVA/Human-wt/JPN/83A001/1983/G2P[4]	-	-	**88.4***	**89.6***	91.1*	**81.1***	**84.6***	**76.7**	**86.4***	**77.6***	**82.1***
RVA/Human-wt/JPN/89Y1520/1989/G2P[4]	-	-	**87.0***	-	91.2*	**80.7***	**85.6***	-	**87.8***	**84.2***	**85.9***
RVA/Human-tc/KEN/D205/1989/G2P[4]	-	-	**86.1**	93.6	-	-	95.2	96.0	96.7	90.1	93.3
RVA/Human-wt/KEN/AK26/1982/G2P[4]	-	-	**86.4**	94.3	92.1	-	92.5	**83.9**	95.3	-	93.7
RVA/Human-tc/JPN/KUN/1980/G2P[4]	-	-	**89.2**	93.7*	93.8	**85.1***	91.8*	**83.0***	92.1*	90.0	91.4
RVA/Human-wt/JPN/86Y1329/1986/G2P[4]	-	-	-	94.1*	-	-	**85.7***	**79.3***	**87.7***	-	-
RVA/Human-wt/BGD/MMC6/2005/G2P[4]	-	-	-	97.6	95.7	-	97.7	-	97.8	**89.3**	-
RVA/Human-wt/BGN/M334/2013/G2P[4]	-	-	-	-	-	-	97.8	-	-	-	-
RVA/Human-wt/USA/LB2764/2005/G2P[4]	-	-	97.3	96.3*	94.5*	93.5*	94.8*	93.7*	93.0	**84.6***	92.5*
RVA/Human-wt/USA/LB2772/2005/G2P[4]	-	-	-	-	-	91.8*	-	-	-	-	-
RVA/Human-wt/AUS/CK20047/2011/G2P[4]	-	-	-	-	-	94.1*	-	-	-	-	-
RVA/Human-wt/AUS/CK20001/1977/G2P[4]	-	-	**89.6**	-	91.4*	-	**89.7**	**81.4**	-	**84.8***	-
RVA/Human-wt/AUS/CK20033/2006/G2P[4]	-	-	-	-	**86.4**	-	-	-	-	-	-
RVA/Human-wt/JPN/B110119/2011/G2P[4]	-	-	-	-	94.5	-	**89.0**	**87.9**	-	**78.0**	-
RVA/Human-wt/JPN/B110056/2011/G2P[4]	-	-	-	-	**85.6**	-	92.4	**89.2**	-	**87.6**	-

RVA/Human-wt/IND/116E3D/1993/G2P[4]	-	-	-	-	-	-	-	-	-	**79.3**	-
RVA/Human-wt/AUS/V233/1999/G2P[4]	-	-	-	-	-	-	-	-	-	**78.2**	-
RVA/Human-wt/USA/2007769964/2007/G2P[4]	-	-	-	-	-	-	**89.7**	-	-	-	-
RVA/Human-wt/ITA/PAI11/1996/G2P[4]	-	-	-	-	-	-	92.1	-	-	90.1	-
RVA/Human-wt/BGD/MMC88/2005/G2P[4]	-	-	97.3	-	**87.2**	98.5	-	-	-	**88.5**	97.1
RVA/Human-wt/BGD/J303/2010/G2P[4]	-	-	-	97.5	**87.0**	98.3	-	-	-	**88.2**	97.3
RVA/Human-wt/CAN/RT128-07/2008/G2P[4]	-	-	93.8	96.1*	94.2*	**87.1***	-	-	-	**69.2***	**73.2***
RVA/Human-wt/BGN/J263/2010/G2P[4]	-	-	93.6	97.5	**87.0**	98.5	98.0	98.7	97.5	96.6	-
RVA/Human-wt/BRA/RJ12225/2006/G2P[4]	-	-	97.3	98.0	96.0*	92.6*	-	-	-	-	-
RVA/Human-wt/BRA/MA14286/2007/G2P[4]	-	-	97.3	98.0	95.8*	91.0*	-	-	-	-	-
RVA/Human-wt/BRA/SC19868/2011/G2P[4]	-	-	-	97.8	94.5*	95.5	-	-	-	-	-
RVA/Human-wt/GHA/Ghan-148/2007/G2P[4]	-	-	-	-	94.2*	**85.5***	-	-	-	-	-
RVA/Human-wt/BGN/J253/2010/G2P[4]	-	-	-	-	93.7	-	-	-	-	-	-
RVA/Human-wt/BRA/ES16238/2009/G2P[4]	-	-	-	96.9	**85.7***	94.3*	-	-	-	-	-
RVA/Human-wt/COD/DRC86/2003/G8P[6]	-	-	96.8	97.6	96.6	96.0	96.6	96.9	97.9	90.8	97.4
RVA/Human-wt/IND/RV09/2009/G9P[4]	-	-	-	-	-	-	98.6	-	-	-	-
RVA/Human-wt/BRA/QUI-154-F1/2010/G12P[6]	-	-	-	-	-	-	94.5*	90.5*	**88.9***	**87.2**	96.3
RVA/Human-wt/BGD/RV161/2000/G12P[6]	-	-	98.0	98.0	96.3	97.4	97.1	97.8	97.9	-	98.2
RVA/Human-wt/CHN/TB-Chen/1996/G2P[4]	-	-	**89.7**	96.2	93.4	**87.1**	96.2	**86.3**	97.4	**89.4**	97.3
RVA/Human-wt/MWI/MW670/1999/G4P[8]	-	**87.6**	-	-	-	-	-	-	-	-	-
RVA/Human-wt/MWI/OP530/1999/G4P[8]	-	**87.6**	-	-	-	-	-	-	-	-	-
RVA/Human-wt/BEL/B1711/2002/G6P[6]	-	-	-	-	**-**	97.1	96.3	97.1	97.8	**89.0**	97.7
RVA/Human-wt/IND/RV11/2011/G9P[4]	-	-	-	-	-	-	98.7	-	-	-	-
RVA/Human-tc/USA/WI61/1983/G9P[8]	-	92.2	-	-	-	-	-	-	-	-	-
RVA/Human-wt/MWI/F45/1987/G9P[8]	-	91.5	-	-	-	-	-	-	-	-	-
RVA/Human-wt/PRY/469/2000/G9P[8]	-	98.1	-	-	-	-	-	-	-	-	-
RVA/Human-wt/THA/CU616-TK/09/2009/G9P[8]	-	96.8	-	-	-	-	-	-	-	-	-
RVA/Human-wt/PHL/L26/1987/G12P[4]	-	-	**86.4***	96.8	-	**76.7***	-	-	95.9	-	-
RVA/Human-tc/KEN/KDH651/2010/G12P[8]	-	95.4	-	-	-	-	-	-	-	-	-
RVA/Human-wt/BRA/RJ12419/2006/G12P[8]	-	97.5	-	-	-	-	-	-	-	-	-

*Nucleotide identity is based on partial ORF sequences.
